# GPX4 Defines an Immune-Cold Phenotype and Poor Prognosis in Resected Lung Adenocarcinoma

**DOI:** 10.32604/or.2026.083840

**Published:** 2026-07-16

**Authors:** Ganxin Wang, Zhongan Liu, Tian Zhou, Boting Yang, Jiaqin Chen, Jing Chen, Kai Huang, Yunqing Xu, Quan Tang, Xiangqian Yin, Guangqin Xiao, Sijia Zhang

**Affiliations:** 1 Cancer Center, Union Hospital, Tongji Medical College, Huazhong University of Science and Technology, Wuhan, China; 2 Department of Infectious Diseases, Union Hospital, Tongji Medical College, Huazhong University of Science and Technology, Wuhan, China; 3 Department of Biophysics, Center for Integrative Physiology and Molecular Medicine (CIPMM), School of Medicine, Saarland University, Homburg, Germany; 4 Department of Biomedical Sciences, Institute for Health Research and Education, Osnabrück University, Osnabrück, Germany; 5 Department of Oncology, People’s Hospital of Huangpi District, Jianghan University, Wuhan, China; 6 Department of Oncology, Hubei Aerospace Hospital, Xiaogan, China

**Keywords:** Lung adenocarcinoma, GPX4, tumor immune microenvironment, PD-L1, nomogram

## Abstract

**Objectives:** Ferroptosis resistance may contribute to tumor progression and immune escape. This study evaluated the prognostic and immunological significance of glutathione peroxidase 4 (GPX4), a core ferroptosis-suppressive enzyme, in surgically resected lung adenocarcinoma. **Methods:** We retrospectively analyzed 104 patients with primary lung adenocarcinoma who underwent curative resection. GPX4 protein expression was assessed by immunohistochemistry (IHC) using the histological score (H-score), and patients were classified as GPX4-low (n = 54) or GPX4-high (n = 50). Intratumoral immune contexture was quantified using CD3, CD4, CD8, CD68, programmed cell death protein 1 (PD-1), and programmed death-ligand 1 (PD-L1) staining. Disease-free survival (DFS) and overall survival (OS) were analyzed using Cox regression. Cutoff sensitivity analyses, category consolidation, ridge-penalized Cox regression, events-per-variable assessment, bootstrap internal validation, and interobserver reproducibility testing were performed to strengthen statistical robustness. **Results:** GPX4-high tumors were associated with systemic inflammatory and immune-related features, including elevated fibrinogen (*p* = 0.015), lower lymphocyte-to-monocyte ratio (*p* = 0.003), and altered aspartate aminotransferase-to-alanine aminotransferase ratio (*p* = 0.028). GPX4-high tumors showed reduced intratumoral CD3^+^, CD4^+^, CD8^+^, and CD68^+^ immune-cell infiltration, together with increased PD-1 and PD-L1 expression, indicating an immune-cold yet checkpoint-enriched phenotype. After category consolidation and ridge-penalized multivariable adjustment, high GPX4 expression remained independently associated with worse DFS (HR, 8.63; 95% CI, 2.99–24.91; *p* < 0.001) and OS (HR, 6.94; 95% CI, 2.44–19.74; *p* < 0.001). GPX4-based prognostic models showed bias-corrected C-index values of 0.782 for DFS and 0.826 for OS, with calibration slopes of 0.964 and 0.937, respectively. **Conclusions:** High GPX4 expression identifies a clinically adverse, ferroptosis-resistant, immune-remodeled phenotype in resected lung adenocarcinoma. Integrating GPX4 with clinicopathological and inflammatory variables may improve postoperative risk stratification.

## 1 Introduction

Postoperative recurrence remains a major clinical challenge in patients with resected lung adenocarcinoma [1,2]. Although pathological staging remains central to risk assessment and treatment decision-making, it does not fully capture the biological heterogeneity of surgically resected tumors [3,4]. Patients with similar anatomical stages can experience markedly divergent clinical courses ranging from durable remission to early relapse and death [4,5]. This variability highlights the need for biologically informative biomarkers that reflect tumor-intrinsic survival programs, immune context, and postoperative recurrence risk beyond conventional clinicopathological parameters [5,6].

Ferroptosis is an iron-dependent form of regulated cell death driven by phospholipid peroxidation and membrane oxidative damage [7,8]. Among the antioxidant systems that inhibit ferroptosis, glutathione peroxidase 4 (GPX4) is a central enzymatic regulator [9]. By reducing phospholipid hydroperoxides to nontoxic lipid alcohols, GPX4 protects cells from lethal lipid peroxidation and preserves membrane integrity [8,9]. In cancer cells exposed to oncogenic stress, metabolic rewiring, hypoxia, inflammation, and anticancer therapy, sustained GPX4 activity may provide a survival advantage by suppressing ferroptotic cell death [10]. Thus, GPX4 is not merely a ferroptosis-related marker but also a potential survival node linking redox homeostasis [11], therapeutic resistance [12], and tumor adaptation [13].

The biological relevance of GPX4 is particularly compelling in lung adenocarcinoma, a disease characterized by molecular heterogeneity, metabolic plasticity, and variable immune responsiveness [14,15,16]. Experimental studies have implicated GPX4 in resistance to platinum-based chemotherapy, epidermal growth factor receptor (EGFR) tyrosine kinase inhibitors, and ferroptosis-inducing agents [17,18]. Suppression or degradation of GPX4 can restore lipid peroxidation and sensitize malignant cells to ferroptotic death, thereby supporting its potential role as a biomarker and therapeutic vulnerability [19,20,21]. Recently, ferroptosis has been recognized as an immunomodulatory process [22]. Ferroptotic tumor cells can influence antigen presentation, inflammatory signaling, dendritic cell activation, macrophage polarization, and cytotoxic T-cell function [23,24]. These observations raise the possibility that GPX4-high tumors may evade immune control not only by resisting ferroptotic death but also by shaping an immune-suppressed tumor microenvironment (TME).

Previous clinical studies have suggested a prognostic role for GPX4 in lung cancer. Notably, Liu et al. [25] reported that GPX4 protein expression, assessed by immunohistochemistry (IHC), was associated with poorer disease-free survival (DFS) and overall survival (OS) in patients with resected non-small cell lung cancer (NSCLC). That study provided important evidence linking GPX4 expression to adverse clinical outcomes. However, several questions remain unanswered. Previous studies primarily established GPX4 as a prognostic marker, but did not comprehensively define its immune context, quantify intratumoral and peritumoral immune-cell infiltration, or clarify its relationship with the programmed cell death protein 1 (PD-1)/programmed death-ligand 1 (PD-L1) axis and systemic inflammatory features [25,26,27]. Therefore, whether GPX4 identifies a biologically coherent immune-cold phenotype in resected lung adenocarcinoma remains unclear.

This question is clinically relevant because the tumor immune microenvironment (TIME) strongly influences recurrence risk and therapeutic response [5]. Tumors enriched in CD8^+^ T cells and other effector immune populations generally reflect active antitumor surveillance, whereas tumors with reduced immune infiltration often display immune-cold or immune-excluded features [28,29]. PD-1 and PD-L1 further shape immune escape and influence responses to immune checkpoint blockade (ICB) [30]. Therefore, a biomarker that links ferroptosis resistance with immune exclusion could provide insights into how tumor-intrinsic survival programs interact with host antitumor immunity. GPX4 is a plausible candidate for this role, but its relationship with spatial immune infiltration and immune-checkpoint expression has not been systematically evaluated at the protein level in surgically resected lung adenocarcinoma.

In the present study, we focused specifically on resected lung adenocarcinoma, rather than broadly generalizing to all NSCLC histologies. This distinction is important because lung adenocarcinoma and lung squamous cell carcinoma differ in their molecular drivers, metabolic dependencies, ferroptosis-related biology, and immune architecture. We assessed GPX4 protein expression by IHC and evaluated its association with clinicopathological features, systemic inflammatory and nutritional indices, DFS, and OS. We further quantified immune-cell infiltration markers and immune-checkpoint expression, including CD3^+^, CD4^+^, CD8^+^, CD68^+^, PD-1-positive, and PD-L1-positive cells, within the TIME.

By integrating GPX4 protein expression, immune profiling, systemic host-related features, and postoperative outcome modeling, this study extends previous work by defining a GPX4-high, immune-cold, checkpoint-enriched phenotype in resected lung adenocarcinoma. In addition, we evaluated the robustness of GPX4-based risk stratification using revised statistical approaches addressing cutoff selection, IHC scoring reproducibility, sparse-event modeling, events-per-variable constraints, and internal validation. Together, these analyses suggest that GPX4 may represent a potential molecular link between ferroptosis resistance, immune escape, and postoperative recurrence risk in lung adenocarcinoma.

## 2 Methods

### 2.1 Study Design

This retrospective observational study included 104 patients with primary lung adenocarcinoma who underwent curative-intent surgical resection at Wuhan Union Hospital, Tongji Medical College, Huazhong University of Science and Technology, between 1 January 2018, and 31 October 2025. Follow-up was administratively censored on 30 November 2025, thereby ensuring that all included patients had at least approximately 1 month of potential follow-up. Formalin-fixed, paraffin-embedded (FFPE) tumor specimens suitable for tissue microarray (TMA) construction and complete GPX4 IHC data were available for all patients. The institutional cohort was restricted to adenocarcinoma-spectrum lung cancer, including adenocarcinoma *in situ* and invasive lung adenocarcinoma. Lung squamous cell carcinoma and other non-adenocarcinoma NSCLC histologies were not included in the primary IHC cohort. Patients were excluded if they had received neoadjuvant therapy, undergone sublobar resection, had a history of another malignancy, or lacked definitive pathological tumor–node–metastasis (TNM) staging information. Tumors were staged according to the 8th edition of the American Joint Committee on Cancer (AJCC) staging system [4]. Survival follow-up was calculated from the date of surgery to recurrence, death, last contact, or administrative censoring, whichever occurred first. The median observed follow-up duration was 39.0 months (range, 1.0–95.0 months), and the median follow-up estimated using the reverse Kaplan–Meier method was 46.0 months. Patients who did not experience an event before the administrative censoring date were treated as right-censored observations in survival analyses. The study protocol was reviewed and approved by the Ethics Committee of Wuhan Union Hospital, Tongji Medical College, Huazhong University of Science and Technology (Approval No. 2021-0737-02). The study was conducted in accordance with the Declaration of Helsinki and relevant institutional requirements. The requirement for written informed consent was waived by the ethics committee because of the retrospective design and the use of de-identified clinical and pathological data.

### 2.2 Data Collection and Data Completeness

Clinicopathological data were retrieved from electronic medical records and pathology reports, including sex, age, Eastern Cooperative Oncology Group (ECOG) performance status, smoking history, body mass index (BMI), tumor laterality, histopathological subtype, pathological T stage, pathological N stage, pathological stage, postoperative high-risk pathological features, and Ki-67 expression. Preoperative laboratory parameters measured within 7 days before surgery were collected, including carcinoembryonic antigen (CEA), neutrophil count, lymphocyte count, monocyte count, platelet count, C-reactive protein (CRP), fibrinogen, albumin, alkaline phosphatase (ALP), alanine aminotransferase (ALT), aspartate aminotransferase (AST), and gamma-glutamyl transferase (γ-GGT). Inflammation- and nutrition-related indices were calculated from the corresponding laboratory measurements, including neutrophil-to-lymphocyte ratio (NLR), lymphocyte-to-monocyte ratio (LMR), platelet-to-lymphocyte ratio (PLR), C-reactive protein-to-albumin ratio (CAR), aspartate aminotransferase-to-platelet ratio index (APRI), and AST-to-ALT ratio (AAR). Data completeness was systematically assessed before analysis. All variables included in the clinicopathological, laboratory, IHC, and survival analyses were available for all 104 patients. No missing data were identified; therefore, no imputation was performed, and all analyses were conducted using the complete dataset.

### 2.3 Follow-up and Outcome Definitions

Patients were followed through medical record review and telephone contact every 3–6 months during the first 2 postoperative years and every 6 months thereafter. Follow-up completeness was systematically verified before survival analysis, and no patients were lost to follow-up. OS was defined as the interval from the date of surgery to death from any cause [31]. Patients who were alive at the end of follow-up were right-censored at the date of last confirmed follow-up or administrative censoring, whichever occurred first. DFS was defined as the interval from the date of surgery to the first documented recurrence, metastasis, or death from any cause [31]. Patients without a DFS event were right-censored at the date of last confirmed disease assessment or administrative censoring, whichever occurred first.

### 2.4 Immunohistochemistry and Digital Image Analysis

IHC was performed on 4-μm FFPE tumor sections using the BOND-III fully automated IHC and *in situ* hybridization (ISH) staining platform (Leica Biosystems, Nussloch, Germany) according to standardized automated staining protocols. Tissue sections were deparaffinized and rehydrated using the BOND system, followed by heat-induced epitope retrieval with BOND Epitope Retrieval Solution 2, an ethylenediaminetetraacetic acid (EDTA)-based retrieval buffer (pH 9.0; Leica Biosystems, Newcastle upon Tyne, UK). Immunodetection was performed using the BOND Polymer Refine Detection System (Leica Biosystems, Newcastle upon Tyne, UK). The primary antibodies were as follows: GPX4 mouse monoclonal antibody, clone 3F5G5, catalog number 67763-1-Ig, working dilution 1:2000 (Proteintech Group, Inc., Rosemont, IL, USA); CD3 epsilon rabbit monoclonal antibody, clone D7A6E, catalog number 85061, working dilution 1:200 (Cell Signaling Technology, Danvers, MA, USA); CD4 rabbit monoclonal antibody, clone EP204, catalog number 48274, working dilution 1:100 (Cell Signaling Technology, Danvers, MA, USA); CD8 alpha mouse monoclonal antibody, clone C8/144B, catalog number 90257, working dilution 1:200 (Cell Signaling Technology, Danvers, MA, USA); CD68 rabbit monoclonal antibody, clone D4B9C, catalog number 76437, working dilution 1:400 (Cell Signaling Technology, Danvers, MA, USA); PD-1 intracellular domain rabbit monoclonal antibody, clone D4W2J, catalog number 86163, working dilution 1:200 (Cell Signaling Technology, Danvers, MA, USA); and PD-L1 rabbit monoclonal antibody, clone E1L3N, catalog number 13684, working dilution 1:400 (Cell Signaling Technology, Danvers, MA, USA). Positive and negative controls were included in each staining run to ensure staining specificity and technical consistency. Negative controls were processed by omitting the primary antibody or using species-matched control immunoglobulin under otherwise identical staining conditions. GPX4 expression in tumor cells was quantified using the histological score (H-score) method, with scores ranging from 0 to 300 [26]. The H-score was calculated as follows: H-score = 1 × percentage of weakly stained tumor cells + 2 × percentage of moderately stained tumor cells + 3 × percentage of strongly stained tumor cells [26]. To characterize the TIME, intratumoral immune-cell densities were quantified using CD3, CD4, CD8, CD68, PD-1, and PD-L1 staining. Whole-slide images were acquired using a Leica digital slide scanner and analyzed using Aperio ImageScope software and the associated image-analysis toolbox (Leica Biosystems Imaging, Inc., Vista, CA, USA). For each case, five representative 1-mm^2^ regions were selected, and the mean density of positively stained CD3, CD4, CD8, CD68, PD-1, and PD-L1 cells was calculated. This standardized workflow enabled integrated assessment of GPX4 expression and local immune contexture within the same surgically resected cohort.

### 2.5 Reproducibility Assessment of GPX4 IHC Scoring

To evaluate the reproducibility of GPX4 IHC scoring, 30 cases were randomly selected from the full cohort of 104 patients and independently reassessed by two observers who were blinded to clinicopathological characteristics, survival outcomes, and original scoring results. Interobserver agreement for continuous GPX4 H-scores was assessed using the intraclass correlation coefficient (ICC) based on an absolute-agreement model. Agreement for dichotomized GPX4 low/high classification using a cutoff of 245 was assessed using Cohen’s κ coefficient. The design and summary of the reproducibility assessment are presented in Supplementary Table S1, and the corresponding reproducibility statistics are presented in Supplementary Fig. S1 and Supplementary Table S2.

### 2.6 Cutoff Determination and Sensitivity Analyses

Optimal cutoffs for GPX4, age, Ki-67, NLR, PLR, LMR, CAR, APRI, and AAR were initially derived using X-tile software, version 3.6.1, according to their associations with DFS and OS. Because outcome-driven cutoff selection can introduce overfitting and upwardly biased estimates of prognostic performance, prespecified sensitivity analyses were conducted using cutoff definitions independent of survival outcomes. Continuous variables were reclassified using cohort-specific median values and tertile-based categories defined by the empirical 33rd and 67th percentiles. Predefined clinical or conventional thresholds were additionally evaluated when they generated analyzable patient strata, including age >60 years, Ki-67 >20%, NLR >3, PLR >150, LMR ≤3, CAR >0.1, and AAR >1.0. The predefined APRI threshold of >0.5 was examined but was not applicable in this cohort because it produced an empty comparison group; therefore, no inferential sensitivity analysis could be performed using this threshold. Because no widely accepted clinical threshold exists for GPX4 expression by H-score, the cohort median was used as the primary non-outcome-based cutoff for sensitivity analyses. The median GPX4 H-score was 245, which yielded the same low- and high-expression distribution as the original X-tile-based classification. To evaluate the robustness of the principal GPX4 findings, GPX4 expression was analyzed as a continuous standardized variable, a median-dichotomized variable, and tertile-based ordinal and categorical variables. The complete set of X-tile-derived cutoffs, median cutoffs, tertile categories, and predefined thresholds is summarized in Supplementary Table S3. GPX4-focused robustness analyses are presented in Supplementary Table S4, and sensitivity analyses for the remaining continuous variables are presented in Supplementary Tables S5 and S6.

### 2.7 Public Transcriptomic Analyses

Public transcriptomic datasets were analyzed as supportive, exploratory resources to examine whether GPX4 transcript abundance was associated with prognosis, immune-related transcriptional features, and immune-cell infiltration. RNA-sequencing data and corresponding clinicopathological information for lung adenocarcinoma were obtained from the Genomic Data Commons (GDC) Data Portal and The Cancer Genome Atlas lung adenocarcinoma project (TCGA-LUAD). The TCGA-LUAD dataset was used as the public transcriptomic reference cohort to maintain histological consistency with the institutional IHC cohort. Database URLs were as follows: GDC Data Portal, https://portal.gdc.cancer.gov/; TCGA-LUAD project, https://portal.gdc.cancer.gov/projects/TCGA-LUAD; Kaplan–Meier Plotter, https://kmplot.com/analysis/index.php?cancer=lung&p=service; Gene Ontology, https://geneontology.org/; and Kyoto Encyclopedia of Genes and Genomes, https://www.genome.jp/kegg/. Survival analyses based on GPX4 mRNA expression were performed using the Kaplan–Meier Plotter lung cancer module. Differential expression analysis was performed in R version 4.6.0 using Bioconductor version 3.23 and the limma package version 3.68.4. Samples were stratified according to median GPX4 expression or immune/stromal scores, as appropriate. Differentially expressed genes were defined using an absolute log^2^ fold change of at least 1.0, corresponding to a minimum two fold difference, together with a nominal *p*-value < 0.05 and a Benjamini–Hochberg false discovery rate (FDR) < 0.05. Genes meeting these criteria were used for downstream functional annotation. Gene Ontology (GO) and Kyoto Encyclopedia of Genes and Genomes (KEGG) enrichment analyses were performed using clusterProfiler version 4.20.0 and org.Hs.eg.db version 3.23.1. Enriched terms were considered statistically significant at an adjusted *p*-value < 0.05 and FDR *q*-value < 0.05. To estimate immune-cell composition from bulk transcriptomic data, the immunedeconv framework version 2.1.4 was applied. This framework integrates established deconvolution algorithms, including Tumor Immune Estimation Resource (TIMER), xCell, Microenvironment Cell Populations-counter (MCP-counter), Cell-type Identification By Estimating Relative Subsets of RNA Transcripts (CIBERSORT), Estimating the Proportions of Immune and Cancer cells (EPIC), and quantification of the tumor immune contexture from human RNA-seq data (quanTIseq). All transcriptomic findings were interpreted cautiously because mRNA abundance may not directly reflect protein expression, post-transcriptional regulation, post-translational modification, or spatially resolved immunohistochemical staining patterns. Accordingly, no direct quantitative comparison was made between TCGA-derived GPX4 mRNA expression and GPX4 immunohistochemical H-scores in the institutional cohort.

### 2.8 Statistical Analysis

Continuous variables were summarized as mean ± standard deviation (SD) or median with interquartile range (IQR), as appropriate; categorical variables were summarized as frequencies and percentages. Between-group comparisons were performed using Student’s *t*-test or Wilcoxon rank-sum test for continuous variables, and *χ^2^* test or Fisher’s exact test for categorical variables. Survival curves were estimated using the Kaplan–Meier method and compared using the log-rank test. Univariable Cox proportional hazards regression was used to assess the associations between candidate variables and DFS or OS. Candidate variables were selected a priori according to their clinical relevance, and included demographic, clinicopathological, laboratory, inflammatory, nutritional, and IHC parameters. Variables associated with the corresponding endpoint in the univariable analysis (*p* < 0.05) were considered for multivariable modeling. To avoid overadjustment and collinearity, biologically overlapping variables were not entered simultaneously. Pathological stage was preferentially used instead of separate T and N categories, and GPX4 expression was retained as the primary biomarker of interest. Because several variables had sparse events or quasi-complete separation in standard Cox models, categories with small subgroup sizes were consolidated before modeling, and ridge-penalized Cox regression with an L2 penalty was used as a sensitivity approach to stabilize coefficient estimation. The events-per-variable (EPV) ratio was calculated for the primary prognostic models. Given the limited number of DFS and OS events, primary predictive models were specified as parsimonious GPX4-based models, including pathological stage and GPX4 expression, whereas expanded ridge-penalized models were interpreted as sensitivity analyses. Correlation analyses were performed using Pearson product–moment correlation coefficients. Pearson correlation was used to evaluate linear associations between continuous quantitative variables, including GPX4 expression, immune-cell densities, immune checkpoint markers, transcriptomic immune scores, and inferred immune-cell fractions. Correlation coefficients are reported as Pearson’s r, with two-sided *p* values. For correlation heatmaps, the color scale represents Pearson’s r, and statistical significance was annotated according to two-sided *p* values. Correlation analyses were considered exploratory and were interpreted in the context of the retrospective study design. Discrimination was assessed using Harrell’s concordance index (C-index) and internal validation was performed using bootstrap resampling to estimate the optimism-corrected C-index and calibration slope. Calibration was evaluated by comparing the predicted and observed 1-, 2-, and 3-year survival rates. Decision curve analysis (DCA) was used to assess potential clinical utility. Time-dependent receiver operating characteristic (ROC) curves and area under the curve (AUC) values were calculated as exploratory performance measures and interpreted using bootstrap-corrected metrics. The proportional hazards assumption was assessed using Schoenfeld residuals and log-minus-log survival plots. All tests were two-sided, and *p* < 0.05 was considered statistically significant. Statistical analyses were performed using SAS software, version 9.4 (SAS Institute, Cary, NC, USA), and R software, version 4.6.0 (R Foundation for Statistical Computing, Vienna, Austria). The R packages used for survival modeling, penalized regression, visualization, correlation analysis, decision curve analysis, and validation included survival version 3.8-6, survminer version 0.5.2, survivalROC version 1.0.3.1, timeROC version 0.4.1, glmnet version 5.0, ggDCA version 1.1, forestplot version 3.2.0, ggplot2 version 4.0.3, pheatmap version 1.0.13, and immunedeconv version 2.1.4. Additional transcriptomic analysis packages are described in Section 2.7.

## 3 Results

### 3.1 Clinicopathological Characteristics of the Study Cohort

The baseline clinicopathological and laboratory characteristics of the 104 patients with surgically resected lung adenocarcinoma are summarized in Table 1. The cohort included 47 men (45.2%) and 57 women (54.8%), and 71 patients (68.3%) were aged ≥ 60 years. Thirty-one patients (29.8%) had a history of previous or current smoking. Most patients had a preserved functional status, with an ECOG performance status of 0 in 62 patients (59.6%) and 1 in 39 (37.5%). Histologically, the cohort comprised of 11 cases of adenocarcinoma *in situ* (10.6%) and 93 cases of invasive lung adenocarcinoma (89.4%). Tumors were located in the left lung in 64 patients (61.5%) and the right lung in 40 patients (38.5%). According to the 8th edition of the AJCC staging system, 71 patients (68.3%) had pathological stage I disease, 12 (11.5%) had stage II disease, and 21 (20.2%) had stage III disease. Postoperative high-risk pathological features were absent in 36 patients (34.6%), whereas 52 patients (50.0%) had a single high-risk feature and 16 patients (15.4%) had multiple high-risk features. The preoperative CEA levels were ≤5 μg/L in 75 patients (72.1%) and >5 μg/L in 29 patients (27.9%).

**Table 1:** Baseline characteristics of patients with surgically resected lung adenocarcinoma.

Characteristic	Category	Overall Cohort, n (%)
Total		104 (100.0)
Sex	Male	47 (45.2)
	Female	57 (54.8)
Age, years	≤60	71 (68.3)
	>60	33 (31.7)
ECOG performance status	0	62 (59.6)
	1	39 (37.5)
	2	3 (2.9)
Smoking history	Smoker	31 (29.8)
	Never smoker	73 (70.2)
BMI category	Underweight	13 (12.5)
	Normal weight	68 (65.4)
	Overweight	23 (22.1)
Pathological T stage	T0	3 (2.9)
	T1	71 (68.3)
	T2	23 (22.1)
	T3	7 (6.7)
Pathological N stage	N0	78 (75.0)
	N1	5 (4.8)
	N2	21 (20.2)
Pathological stage	I	71 (68.3)
	II	12 (11.5)
	III	21 (20.2)
Tumor laterality	Left lung	64 (61.5)
	Right lung	40 (38.5)
Histopathological subtype	Adenocarcinoma *in situ*	11 (10.6)
	Invasive adenocarcinoma	93 (89.4)
Post-op high-risk features	None	36 (34.6)
	Single	52 (50.0)
	Multiple	16 (15.4)
Ki-67 expression by IHC, %	≤20	84 (80.8)
	>20–40	14 (13.5)
	>40	6 (5.8)
GPX4 expression by IHC	Low	54 (51.9)
	High	50 (48.1)
CEA, μg/L	≤5	75 (72.1)
	>5	29 (27.9)
Neutrophil count, ×10^9^/L	≤1.8	3 (2.9)
	>1.8–6.3	98 (94.2)
	>6.3	3 (2.9)
Lymphocyte count, ×10^9^/L	≤1.1	13 (12.5)
	>1.1–3.2	91 (87.5)
Monocyte count, ×10^9^/L	0.1–0.6	95 (91.3)
	>0.6	9 (8.7)
Platelet count, ×10^9^/L	≤125	9 (8.7)
	>125–350	90 (86.5)
	>350	5 (4.8)
CRP, mg/L	≤4	85 (81.7)
	>4	19 (18.3)
Fibrinogen, g/L	≤2.0	5 (4.8)
	>2.0–4.0	89 (85.6)
	>4.0	10 (9.6)
Albumin, g/L	≤35	10 (9.6)
	>35	94 (90.4)
ALP, U/L	≤40	2 (1.9)
	>40–150	101 (97.1)
	>150	1 (1.0)
ALT, U/L	5–40	98 (94.2)
	>40	6 (5.8)
AST, U/L	8–40	101 (97.1)
	>40	3 (2.9)
γ-GGT, U/L	≤11	10 (9.6)
	>11–50	86 (82.7)
	>50	8 (7.7)
NLR	≤2.30	64 (61.5)
	>2.30–3.34	25 (24.0)
	>3.34	15 (14.4)
PLR	≤110.53	40 (38.5)
	>110.53–180.81	54 (51.9)
	>180.81	10 (9.6)
LMR	≤3.12	19 (18.3)
	>3.12–4.69	38 (36.5)
	>4.69	47 (45.2)
CAR	≤0.03	48 (46.2)
	>0.03–0.17	44 (42.3)
	>0.17	12 (11.5)
APRI	≤0.10	46 (44.2)
	>0.10–0.13	22 (21.2)
	>0.13	36 (34.6)
AAR	≤0.69	28 (26.9)
	>0.69–0.87	36 (34.6)
	>0.87	40 (38.5)

Note: ECOG, Eastern Cooperative Oncology Group; BMI, body mass index; IHC, immunohistochemistry; CEA, carcinoembryonic antigen; CRP, C-reactive protein; ALP, alkaline phosphatase; ALT, alanine aminotransferase; AST, aspartate aminotransferase; γ-GGT, gamma-glutamyl transferase; NLR, neutrophil-to-lymphocyte ratio; PLR, platelet-to-lymphocyte ratio; LMR, lymphocyte-to-monocyte ratio; CAR, C-reactive protein-to-albumin ratio; APRI, AST to platelet ratio index; AAR, AST to ALT ratio.

### 3.2 Association between GPX4 Expression and Clinicopathological and Laboratory Features

**Figure 1:**
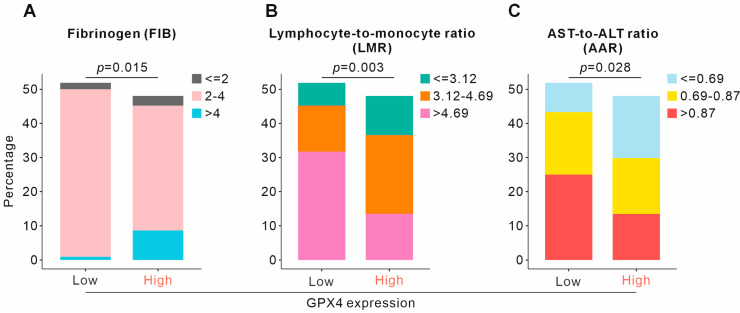
**Distribution of selected systemic laboratory indices according to GPX4 expression status**. Stacked bar plots show the proportional distribution of categorized fibrinogen (FIB), lymphocyte-to-monocyte ratio (LMR), and AST-to-ALT ratio (AAR) across GPX4-low and GPX4-high tumors. (**A**) FIB categories were defined as ≤2.0, >2.0–4.0, and >4.0 g/L. (**B**) LMR categories were defined as ≤3.12, >3.12–4.69, and >4.69. (**C**) AAR categories were defined as ≤0.69, >0.69–0.87, and >0.87. Bar heights indicate the percentage of patients within each GPX4 expression group, and colors denote the corresponding laboratory categories. Associations between GPX4 expression status and categorized variables were assessed using the *χ^2^* test or Fisher’s exact test, as appropriate. All tests were two-sided, and exact *p*-values are shown above each panel.

To define the clinical and biological correlates of GPX4 protein expression, patients were stratified into GPX4-low and GPX4-high groups using a predefined H-score cutoff. As shown in Table 2, GPX4 expression was selectively associated with systemic laboratory features reflecting coagulation, immune cell balance, and metabolic status. GPX4-high tumors were more frequently observed in patients with elevated fibrinogen levels; specifically, fibrinogen >4.0 g/L was present in 9 of 50 patients in the GPX4-high group compared to 1 of 54 patients in the GPX4-low group, indicating a positive association between GPX4 expression and a procoagulant inflammatory phenotype (Fig. 1A). GPX4 expression is also associated with LMR distribution. Patients with GPX4-high tumors were enriched in the lower and intermediate LMR categories, whereas the highest LMR category was more frequent in the GPX4-low group, suggesting that elevated GPX4 expression is linked to reduced lymphocyte predominance in the systemic immune profile (Fig. 1B). In addition, GPX4-high expression was associated with lower AAR categories; AAR ≤0.69 was observed in 38.0% of GPX4-high cases compared with 16.7% of GPX4-low cases, whereas AAR >0.87 was more frequent in the GPX4-low group (Fig. 1C). Together, these findings indicate that GPX4 expression in resected lung adenocarcinoma is not primarily explained by conventional staging parameters, but is selectively associated with systemic coagulation–inflammatory activation, altered peripheral immune balance, and metabolic laboratory features.

**Table 2:** Associations between GPX4 expression and baseline clinicopathological and laboratory characteristics in patients with lung adenocarcinoma.

Variable	Category	Low (n = 54), n (%)	High (n = 50), n (%)	*p*
Sex	Male	22 (40.7)	25 (50.0)	0.343
	Female	32 (59.3)	25 (50.0)	
Age, years	≤60	38 (70.4)	33 (66.0)	0.632
	>60	16 (29.6)	17 (34.0)	
ECOG	0	35 (64.8)	27 (54.0)	0.486
	1	18 (33.3)	21 (42.0)	
	2	1 (1.9)	2 (4.0)	
Smoking history	Never smoker	42 (77.8)	31 (62.0)	0.079
	Smoker	12 (22.2)	19 (38.0)	
BMI category	Underweight	6 (11.1)	7 (14.0)	0.78
	Normal weight	37 (68.5)	31 (62.0)	
	Overweight or obese	11 (20.4)	12 (24.0)	
T stage	T0	1 (1.9)	2 (4.0)	0.264
	T1	34 (63.0)	37 (74.0)	
	T2	16 (29.6)	7 (14.0)	
	T3	3 (5.6)	4 (8.0)	
N stage	N0	38 (70.4)	40 (80.0)	0.345
	N1	4 (7.4)	1 (2.0)	
	N2	12 (22.2)	9 (18.0)	
Pathological stage	I	33 (61.1)	38 (76.0)	0.163
	II	9 (16.7)	3 (6.0)	
	III	12 (22.2)	9 (18.0)	
Tumor laterality	Right lung	23 (42.6)	17 (34.0)	0.368
	Left lung	31 (57.4)	33 (66.0)	
Histopathological subtype	Adenocarcinoma *in situ*	4 (7.4)	7 (14.0)	0.275
	Invasive adenocarcinoma	50 (92.6)	43 (86.0)	
Post-op high-risk features	None	22 (40.7)	14 (28.0)	0.269
	Single	26 (48.1)	26 (52.0)	
	Multiple	6 (11.1)	10 (20.0)	
CEA, μg/L	≤5	40 (74.1)	35 (70.0)	0.643
	>5	14 (25.9)	15 (30.0)	
Neutrophil count, ×10^9^/L	≤1.8	1 (1.9)	2 (4.0)	0.713
	>1.8–6.3	51 (94.4)	47 (94.0)	
	>6.3	2 (3.7)	1 (2.0)	
Lymphocyte count, ×10^9^/L	≤1.1	7 (13.0)	6 (12.0)	0.882
	>1.1–3.2	47 (87.0)	44 (88.0)	
Monocyte count, ×10^9^/L	0.1–0.6	51 (94.4)	44 (88.0)	0.243
	>0.6	3 (5.6)	6 (12.0)	
Platelet count, ×10^9^/L	≤125	3 (5.6)	6 (12.0)	0.415
	>125–350	49 (90.7)	41 (82.0)	
	>350	2 (3.7)	3 (6.0)	
CRP, mg/L	≤4	46 (85.2)	39 (78.0)	0.343
	>4	8 (14.8)	11 (22.0)	
Fibrinogen, g/L	≤2.0	2 (3.7)	3 (6.0)	0.015
	>2.0–4.0	51 (94.4)	38 (76.0)	
	>4.0	1 (1.9)	9 (18.0)	
Albumin, g/L	≤35	4 (7.4)	6 (12.0)	0.427
	>35	50 (92.6)	44 (88.0)	
ALP, U/L	≤40	2 (3.7)	0 (0.0)	0.23
	>40–150	52 (96.3)	49 (98.0)	
	>150	0 (0.0)	1 (2.0)	
ALT, U/L	5–40	53 (98.1)	45 (90.0)	0.075
	>40	1 (1.9)	5 (10.0)	
AST, U/L	8–40	53 (98.1)	48 (96.0)	0.513
	>40	1 (1.9)	2 (4.0)	
γ-GGT, U/L	≤11	6 (11.1)	4 (8.0)	0.064
	>11–50	47 (87.0)	39 (78.0)	
	>50	1 (1.9)	7 (14.0)	
Ki-67 expression by IHC, %	≤20	47 (87.0)	37 (74.0)	0.24
	>20–40	5 (9.3)	9 (18.0)	
	>40	2 (3.7)	4 (8.0)	
NLR	≤2.30	38 (70.4)	26 (52.0)	0.157
	>2.30–3.34	10 (18.5)	15 (30.0)	
	>3.34	6 (11.1)	9 (18.0)	
PLR	≤110.53	25 (46.3)	15 (30.0)	0.218
	>110.53–180.81	25 (46.3)	29 (58.0)	
	>180.81	4 (7.4)	6 (12.0)	
LMR	≤3.12	7 (13.0)	12 (24.0)	0.003
	>3.12–4.69	14 (25.9)	24 (48.0)	
	>4.69	33 (61.1)	14 (28.0)	
CAR	≤0.03	28 (51.9)	20 (40.0)	0.284
	>0.03–0.17	22 (40.7)	22 (44.0)	
	>0.17	4 (7.4)	8 (16.0)	
APRI	≤0.10	22 (40.7)	24 (48.0)	0.085
	>0.10–0.13	16 (29.6)	6 (12.0)	
	>0.13	16 (29.6)	20 (40.0)	
AAR	≤0.69	9 (16.7)	19 (38.0)	0.028
	>0.69–0.87	19 (35.2)	17 (34.0)	
	>0.87	26 (48.1)	14 (28.0)	

Note: ECOG, Eastern Cooperative Oncology Group; BMI, body mass index; IHC, immunohistochemistry; CEA, carcinoembryonic antigen; CRP, C-reactive protein; ALP, alkaline phosphatase; ALT, alanine aminotransferase; AST, aspartate aminotransferase; γ-GGT, gamma-glutamyl transferase; NLR, neutrophil-to-lymphocyte ratio; PLR, platelet-to-lymphocyte ratio; LMR, lymphocyte-to-monocyte ratio; CAR, C-reactive protein-to-albumin ratio; APRI, AST to platelet ratio index; AAR, AST to ALT ratio.

### 3.3 GPX4 Is Independently Associated with Postoperative Survival

**Figure 2:**
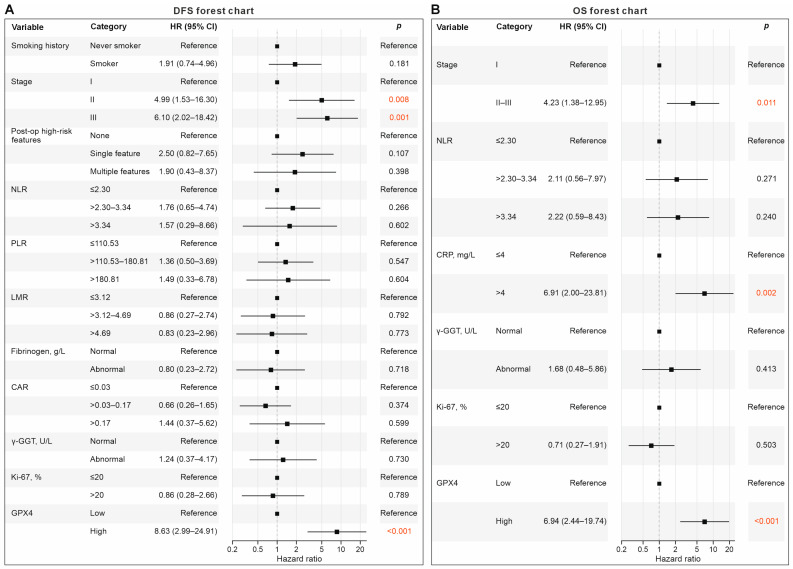
**Multivariable forest plots for postoperative survival after category consolidation and ridge-penalized Cox regression**. (A) Adjusted hazard ratios (HRs) and 95% confidence intervals (CIs) for disease-free survival (DFS). (B) Adjusted HRs and 95% CIs for overall survival (OS). Variables with sparse categories were consolidated before modeling to improve estimate stability. Squares represent point estimates, horizontal lines indicate 95% CIs, and the vertical dashed line denotes HR = 1. Reference categories are indicated as “Reference”. Two-sided *p*-values are shown in the rightmost column, with statistically significant values highlighted in red. Note: CRP, C-reactive protein; γ-GGT, gamma-glutamyl transferase; NLR, neutrophil-to-lymphocyte ratio; PLR, platelet-to-lymphocyte ratio; LMR, lymphocyte-to-monocyte ratio; CAR, C-reactive protein-to-albumin ratio.

To address sparse subgroup events and improve the stability of model estimates, we re-evaluated postoperative survival using category-consolidated ridge-penalized Cox regression [32]. In the DFS model, the pathological stage and GPX4 expression were independently associated with recurrence risk. Compared with stage I disease, stage II and stage III disease were associated with progressively higher risks of DFS events, with adjusted HRs of 4.99 (95% CI, 1.53–16.30; *p* = 0.008) and 6.10 (95% CI, 2.02–18.42; *p* = 0.001), respectively. Notably, high GPX4 expression showed a strong independent association with adverse DFS, with an adjusted HR of 8.63 (95% CI, 2.99–24.91; *p* < 0.001) compared with low GPX4 expression (Fig. 2A and Supplementary Table S7). In the OS model, the pathological stage, CRP level, and GPX4 expression were independent prognostic factors. Patients with stage II–III disease had a significantly increased risk of death compared to those with stage I disease (HR, 4.23; 95% CI, 1.38–12.95; *p* = 0.011), and an elevated CRP level (>4 mg/L) was independently associated with poorer OS (HR, 6.91; 95% CI, 2.00–23.81; *p* = 0.002). Consistent with the DFS analysis, GPX4-high tumors remained associated with substantially worse OS after penalized multivariate adjustment (HR, 6.94; 95% CI, 2.44–19.74; *p* < 0.001; Fig. 2B and Supplementary Table S8). Collectively, these stabilized multivariable models identified GPX4 as a robust adverse prognostic biomarker for both DFS and OS in resected lung adenocarcinoma, independent of pathological stage and selected systemic inflammatory or clinicopathological variables.

### 3.4 GPX4 Upregulation Is Linked to Adverse Prognosis, Ferroptosis-Related Pathway Enrichment, and Immune Remodeling

**Figure 3:**
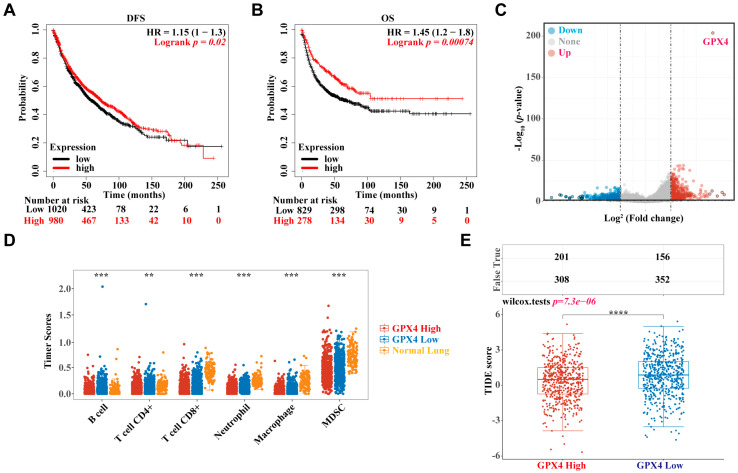
**Exploratory transcriptomic analyses of GPX4 expression, survival and immune-related features**. (**A**,**B**) Kaplan–Meier survival curves stratified by GPX4 mRNA expression for disease-free survival (DFS; (**A**)) and overall survival (OS; (**B**)). Hazard ratios (HRs), log-rank *p*-values and numbers at risk are shown in each panel. (**C**) Volcano plot of differentially expressed genes between GPX4-high and GPX4-low samples. Genes with increased or decreased expression are indicated in red and blue, respectively; non-significant genes are shown in grey. (**D**) Distribution of inferred immune-cell scores across GPX4-high tumors, GPX4-low tumors and normal lung tissues. Immune-cell infiltration scores were estimated from bulk transcriptomic data. (**E**) TIDE-based immune evasion analysis according to GPX4 expression status, including predicted response classification and TIDE score distribution. Box plots show the median, interquartile range and whiskers; statistical comparisons were performed using Wilcoxon tests unless otherwise specified. **p < 0.01, ***p < 0.001, ****p < 0.0001.

To complement the protein-level IHC findings, we performed exploratory transcriptomic analyses to examine whether GPX4 mRNA expression was linked to clinical trajectories, biological pathways, and immune-related features. Sankey visualizations showed the distribution of GPX4 expression across age, sex, pathological stage, and survival status, and further illustrated its relationship with postoperative disease events, smoking history, radiotherapy status, and outcome (Fig. 2A,B). Pathway enrichment analysis was reinterpreted using an adjusted *p* value < 0.05 as the threshold for statistical significance. Genes positively associated with GPX4 showed significant enrichment mainly in the coronavirus disease–COVID-19 and tight junction pathways. By contrast, genes negatively associated with GPX4 were significantly enriched across multiple immune-, stromal-, and extracellular matrix-associated pathways, including interleukin-17 signaling, cytokine–cytokine receptor interaction, extracellular matrix–receptor interaction, focal adhesion, phosphoinositide 3-kinase–protein kinase B signaling, Hippo signaling, AGE–RAGE signaling, and relaxin signaling pathways. These negatively enriched pathways were consistent with the reduced immune-cell infiltration and altered tumor–stromal interaction patterns observed in GPX4-high tumors (Supplementary Fig. S2C,D). In public transcriptomic cohorts, GPX4 mRNA expression stratified both DFS and OS, supporting its prognostic relevance at the transcript level while recognizing that mRNA abundance is not directly equivalent to IHC-based protein expression (Fig. 3A,B). Differential expression analysis further identified a GPX4-associated transcriptional program, with GPX4 being among the most prominently upregulated genes in the GPX4-high group (Fig. 3C). Immune deconvolution demonstrated broad differences in the inferred immune cell scores, including B cells, CD4^+^ and CD8^+^ T cells, neutrophils, macrophages, and myeloid-derived suppressor cells, across GPX4-high tumors, GPX4-low tumors, and normal lung tissues (Fig. 3D). Tumor Immune Dysfunction and Exclusion (TIDE) analysis further suggested that GPX4 expression was associated with altered immune evasion features and predicted immunotherapy responsiveness (Fig. 3E). Together, these exploratory transcriptomic data support a biological link between GPX4, ferroptosis-related metabolic regulation, and immune remodeling, providing an external hypothesis-generating framework for IHC-based observations in resected lung adenocarcinoma.

### 3.5 GPX4-High Lung Adenocarcinoma Is Associated with Immune Exclusion, Checkpoint Enrichment, and Inferior Postoperative Survival

**Figure 4:**
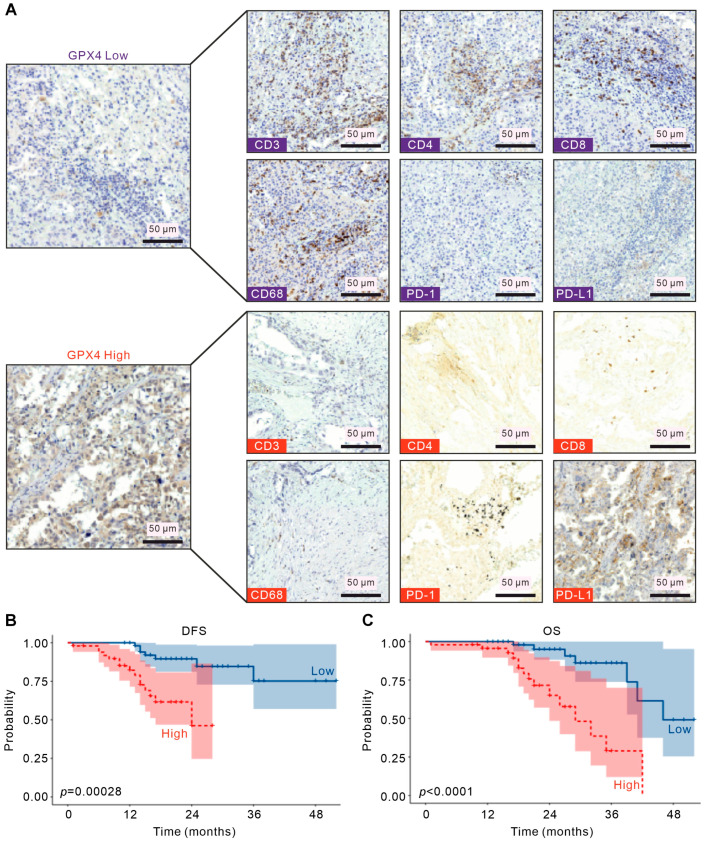
**Spatial immune contexture and postoperative survival according to GPX4 expression in lung adenocarcinoma**. (**A**) Representative immunohistochemical images showing GPX4-low and GPX4-high lung adenocarcinoma tissues and the corresponding immune-marker staining patterns, including CD3, CD4, CD8, CD68, PD-1, and PD-L1. GPX4 immunoreactivity was predominantly cytoplasmic in tumor cells, with low and high expression defined according to staining intensity and H-score assessment. Brown chromogenic signal indicates positive staining, and hematoxylin was used for nuclear counterstaining. Scale bars, 50 μm. (**B**,**C**) Kaplan–Meier curves for disease-free survival (DFS; (**B**)) and overall survival (OS; (**C**)) stratified by GPX4 expression status. Shaded areas represent confidence intervals, and tick marks indicate censored observations. Two-sided log-rank *p*-values are shown.

**Figure 5:**
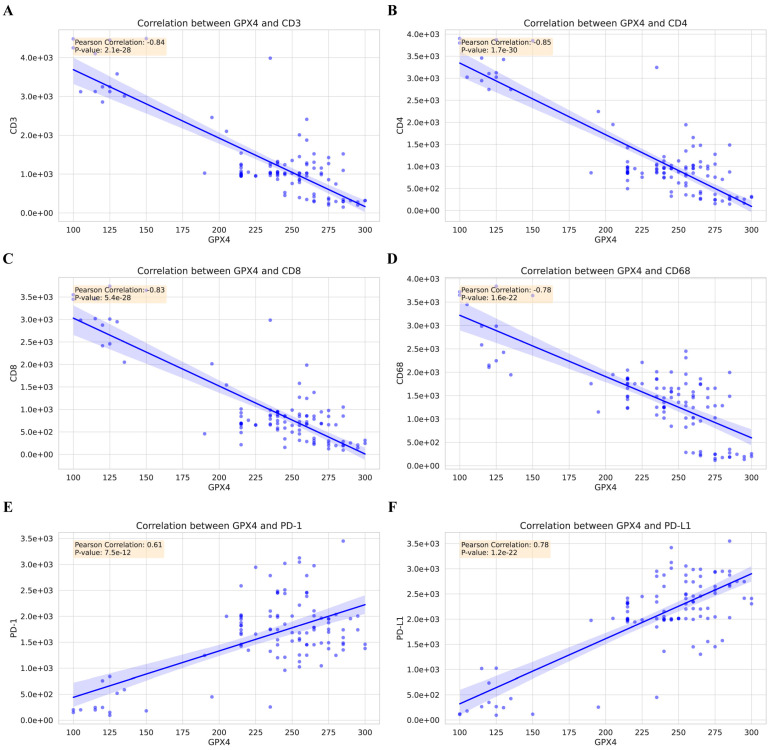
**Correlation between GPX4 expression and intratumoral immune contexture in lung adenocarcinoma**. (**A**–**F**) Scatter plots show the associations between GPX4 H-score and intratumoral immune-marker densities for CD3 (**A**), CD4 (**B**), CD8 (**C**), CD68 (**D**), PD-1 (**E**), and PD-L1 (**F**). Each point represents an individual tumor specimen. Blue lines indicate fitted linear regression trends, and shaded areas denote the corresponding 95% confidence intervals. Correlation coefficients and two-sided *p* values are shown in each panel.

IHC profiling first established that GPX4 expression in lung adenocarcinoma exhibited a graded staining spectrum, ranging from negative to weak, moderate, and strong intensities, with predominant cytoplasmic localization in tumor cells (Supplementary Fig. S3). We then assessed whether this ferroptosis-resistance marker was linked to the spatial immune context of the tumour microenvironment. Representative IHC images showed that GPX4-low tumors were accompanied by dense intratumoral infiltration of CD3^+^, CD4^+^, CD8^+^, and CD68^+^ immune cells, whereas GPX4-high tumors displayed attenuated lymphoid and macrophage infiltration together with stronger PD-1 and PD-L1 staining signals (Fig. 4A). This immune phenotype was clinically relevant because patients with GPX4-high tumors had significantly shorter DFS and OS than those with GPX4-low tumors (Fig. 4B,C). Quantitative immune-marker density analyses further confirmed that GPX4-high tumors had significantly lower intratumoral CD3^+^, CD4^+^, CD8^+^, and CD68^+^ cell densities, but higher PD-1^+^ and PD-L1^+^ cell densities, indicating an immune-depleted yet checkpoint-enriched microenvironment (Supplementary Fig. S4). Consistently, correlation analyses demonstrated inverse associations between GPX4 expression and effector immune cell infiltration and positive associations between GPX4 expression and PD-1/PD-L1 expression (Fig. 5). Additional integrated analyses further linked GPX4 expression to systemic inflammatory and immune-related features: GPX4-high tumors were associated with a lower LMR, GPX4 expression showed a negative correlation with AAR, and the correlation heatmap confirmed that GPX4 clustered inversely with CD3, CD4, CD8, and CD68 but positively correlated with PD-1 and PD-L1 (Supplementary Fig. S5). Together, these findings suggest that high GPX4 expression identifies a biologically coherent subgroup of resected lung adenocarcinomas, characterized by cytoplasmic GPX4 accumulation, reduced antitumor immune infiltration, enhanced immune checkpoint expression, systemic immune imbalance, and adverse postoperative outcomes.

### 3.6 GPX4-Based Nomograms Provide Individualized Postoperative Risk Stratification

**Figure 6:**
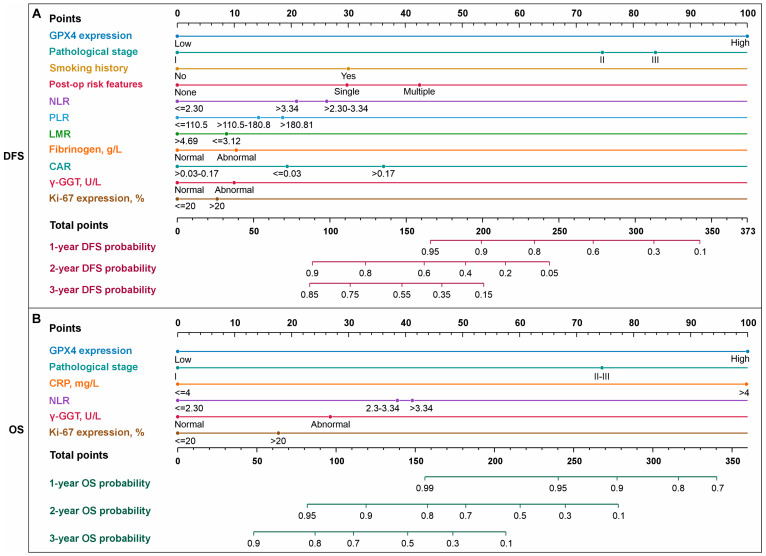
**GPX4-based nomograms for individualized prediction of postoperative survival in lung adenocarcinoma**. (**A**) Nomogram for estimating 1-, 2-, and 3-year disease-free survival (DFS) probability based on the final category-collapsed ridge-penalized multivariable Cox model, including GPX4 expression, pathological stage, smoking history, postoperative high-risk pathological features, NLR, PLR, LMR, fibrinogen, CAR, γ-GGT, and Ki-67 expression. (**B**) Nomogram for estimating 1-, 2-, and 3-year overall survival (OS) probability based on the corresponding final multivariable model, including GPX4 expression, pathological stage, CRP, NLR, γ-GGT, and Ki-67 expression. For each predictor, the patient-specific category is projected onto the upper points scale to assign a point value; the sum of all points is then mapped onto the corresponding 1-, 2-, and 3-year survival probability scales.

GPX4-based nomograms were generated to estimate individualized 1-, 2-, and 3-year DFS and OS probabilities in patients with resected lung adenocarcinoma (Fig. 6A,B). Model stability was supported by the EPV assessment, with 32 DFS events and 22 OS events corresponding to EPV values of 16 and 11, respectively, in the compact GPX4-centered prognostic models (Supplementary Tables S9 and S10). Calibration plots showed acceptable agreement between the predicted and observed 1-, 2-, and 3-year DFS and OS probabilities, with wider uncertainty at later time points, reflecting the limited number of events (Supplementary Fig. S6A–C,G–I). Time-dependent ROC curves demonstrated a high apparent discriminatory ability for DFS and OS at 1, 2, and 3 years (Supplementary Fig. S6D,J), and the dynamic AUC curves generally showed sustained discrimination across follow-up (Supplementary Fig. S6E,K). Because very high apparent AUC values may overestimate performance in a small retrospective cohort, bootstrap internal validation was used to quantify optimism [33]. The apparent C-index was 0.799 and 0.838 for DFS and OS, respectively. After optimism correction, the bias-corrected C-index remained 0.782 for DFS and 0.826 for OS. The corresponding bias-corrected calibration slopes were 0.964 and 0.937, respectively, indicating limited overfitting and preserved calibration after the internal validation (Supplementary Table S11). DCA further indicated that the nomogram-based strategy provided a potential net clinical benefit across selected threshold probabilities compared to the treat-all or treat-none strategies (Supplementary Fig. S6F,L). Overall, these results support a compact GPX4-based prognostic framework for postoperative risk stratification of resected lung adenocarcinomas, indicating that external validation is necessary before clinical implementation.

## 4 Discussion

In this study, we identified GPX4 as a clinically relevant ferroptosis-associated biomarker in surgically resected lung adenocarcinoma. High GPX4 expression was associated with inferior postoperative survival, reduced intratumoral immune-cell infiltration, and increased PD-1/PD-L1 expression, suggesting that GPX4 may mark a biologically distinct tumor state characterized by ferroptosis resistance and immune remodeling. These findings extend the relevance of GPX4 beyond its canonical antioxidant function and support its potential utility for postoperative risk stratification.

The biological rationale for focusing on GPX4 is strong. As a central suppressor of ferroptosis, GPX4 detoxifies phospholipid hydroperoxides and protects tumor cells from lethal lipid peroxidation [9,34,35]. In lung adenocarcinoma, where oncogenic stress, metabolic plasticity, hypoxia, oxidative injury, and therapy resistance shape tumor evolution, sustained GPX4 activity may confer a survival advantage by limiting ferroptotic vulnerability [36,37]. GPX4 may also influence antitumor immunity, as ferroptosis can modulate antigen presentation, inflammatory signaling, dendritic-cell activation, macrophage behavior, and cytotoxic T-cell function [37]. Thus, GPX4 represents a plausible molecular link between tumor-intrinsic stress adaptation and immune evasion.

Our findings are consistent with previous clinical evidence showing that GPX4 protein expression is associated with poor survival in resected NSCLC, particularly in lung adenocarcinoma [25]. However, the present study extends prior work in several important respects. First, our institutional cohort was restricted to lung adenocarcinoma, thereby avoiding overgeneralization across heterogeneous NSCLC histologies. Second, we integrated GPX4 protein expression with spatial immune profiling, systemic inflammatory indices, and postoperative outcome modeling. Third, we found that GPX4-high tumors were not simply characterized by more advanced conventional staging, but instead exhibited an immune-remodeled phenotype marked by reduced effector immune infiltration and enhanced immune-checkpoint expression.

The coexistence of reduced immune-cell infiltration and increased PD-1/PD-L1 expression requires careful interpretation. Although checkpoint expression is often associated with inflamed tumors, PD-1/PD-L1 upregulation can also occur in non-classically inflamed or immune-excluded contexts [38,39]. Potential mechanisms include tumor-intrinsic oncogenic signaling, oxidative stress, hypoxia, stromal remodeling, residual dysfunctional immune-cell activation, and compensatory immune escape [40,41,42]. Accordingly, GPX4-high tumors may not represent a purely immune-desert phenotype, but rather an immune-dysregulated state in which effector-cell entry is limited while checkpoint signaling persists within residual immune or tumor compartments [40,43,44]. Mechanistically, GPX4-mediated suppression of lipid peroxidation may reduce immunogenic ferroptotic signals, alter myeloid-cell function, impair antitumor immune recruitment, and promote a microenvironment permissive to immune evasion [44].

Several analytical strategies strengthened the robustness of this study. Because X-tile-derived outcome-based cutoffs can introduce overfitting and optimistic bias, we performed sensitivity analyses using median-based, tertile-based, continuous-variable, and clinically predefined thresholds. The persistence of the GPX4 survival association across these alternative definitions supports the stability of the principal finding, although independent validation of the cutoff remains necessary. We also assessed GPX4 IHC scoring reproducibility, consolidated sparse categories, applied ridge-penalized Cox regression to reduce unstable estimates, considered EPV constraints, and performed bootstrap internal validation for prognostic models. These steps improved statistical credibility, but they cannot substitute for external validation.

Several limitations should be acknowledged. First, this was a retrospective, single-center study with a modest sample size of 104 patients and a limited number of DFS and OS events. Selection bias is possible because inclusion required available tissue material and complete GPX4 IHC data. Second, the cohort was limited to lung adenocarcinoma; therefore, the findings should not be generalized to lung squamous cell carcinoma or other NSCLC subtypes. Third, public transcriptomic analyses were exploratory, as TCGA-derived GPX4 mRNA abundance is not directly equivalent to protein-level IHC expression and lacks spatial resolution. Finally, the GPX4 cutoff, immune associations, and nomogram performance require validation in larger multicenter cohorts with standardized staining protocols, external calibration, and ideally prospective follow-up.

## 5 Conclusions

**Figure 7:**
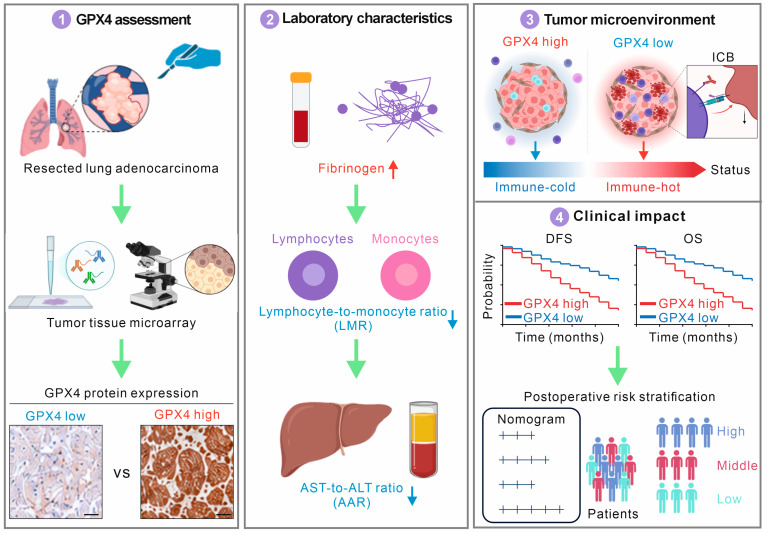
**Schematic illustration summarizing the study design and principal findings**. (1) GPX4 protein expression was assessed in surgically resected lung adenocarcinoma specimens using immunohistochemistry (IHC) on tumor tissue microarrays and quantified by H-score, enabling stratification into GPX4-low and GPX4-high tumors. (2) GPX4-high tumors were associated with systemic laboratory features suggestive of coagulation–inflammatory activation and altered host immune–metabolic status, including increased fibrinogen, reduced lymphocyte-to-monocyte ratio and altered aspartate aminotransferase-to-alanine aminotransferase ratio. (3) GPX4-high tumors displayed an immune-cold tumor microenvironment, characterized by reduced immune-cell infiltration and relative enrichment of immune-checkpoint features, whereas GPX4-low tumors showed a more immune-inflamed phenotype. (4) High GPX4 expression was associated with inferior disease-free and overall survival and supported individualized postoperative risk stratification when incorporated into prognostic nomogram models.

High GPX4 expression identifies a clinically adverse, ferroptosis-resistant, and immune-remodeled phenotype in resected lung adenocarcinoma, characterized by reduced antitumor immune infiltration, immune-checkpoint enrichment, and inferior postoperative survival. By integrating GPX4 protein assessment with immune profiling and survival modeling, this study provides a translational framework linking ferroptosis resistance to immune escape and postoperative recurrence risk. Future multicenter studies should validate GPX4-based risk stratification and determine whether ferroptosis-targeted strategies can enhance antitumor immune responsiveness in GPX4-high lung adenocarcinoma (Fig. 7).

## Data Availability

The authors confirm that the data supporting the findings of this study are available within the article or in the Supplementary Materials.

## Supplementary Materials

The supplementary material is available online at https://www.techscience.com/doi/10.32604/or.2026.083840/s1. Supplementary Figs. S1–S6 and Supplementary Tables S1–S11 provide the supporting experimental data, extended analyses, and source information. Full details are provided in the Supplementary Materials section.

## Abbreviations

AAR	AST-to-ALT ratio/aspartate aminotransferase-to-alanine aminotransferase ratio
AJCC	American Joint Committee on Cancer
ALP	Alkaline phosphatase
ALT	Alanine aminotransferase
APRI	Aspartate aminotransferase-to-platelet ratio index
AST	Aspartate aminotransferase
AUC	Area under the curve
BMI	Body mass index
CAR	C-reactive protein-to-albumin ratio
CEA	Carcinoembryonic antigen
CI	Confidence interval
CIBERSORT	Cell-type Identification By Estimating Relative Subsets of RNA Transcripts
C-index	Concordance index
CRP	C-reactive protein
CSC	China Scholarship Council
DCA	Decision curve analysis
DFS	Disease-free survival
ECOG	Eastern Cooperative Oncology Group
ECM	Extracellular matrix
EDTA	Ethylenediaminetetraacetic acid
EGFR	Epidermal growth factor receptor
EPIC	Estimating the Proportions of Immune and Cancer cells
EPV	Events per variable
FFPE	Formalin-fixed, paraffin-embedded
FIB	Fibrinogen
GDC	Genomic Data Commons
GO	Gene Ontology
GPX4	Glutathione peroxidase 4
γ-GGT	Gamma-glutamyl transferase
HR	Hazard ratio
H-score	Histological score
ICB	Immune checkpoint blockade
ICC	Intraclass correlation coefficient
IHC	Immunohistochemistry
IL-17	Interleukin-17
INR	International normalized ratio
IQR	Interquartile range
ISH	*In situ* hybridization
KEGG	Kyoto Encyclopedia of Genes and Genomes
LMR	Lymphocyte-to-monocyte ratio
MCP-counter	Microenvironment Cell Populations-counter
mRNA	Messenger RNA
NLR	Neutrophil-to-lymphocyte ratio
NSCLC	Non-small cell lung cancer
OS	Overall survival
PD-1	Programmed cell death protein 1
PD-L1	Programmed death-ligand 1
PI3K–Akt	Phosphoinositide 3-kinase–protein kinase B
PLR	Platelet-to-lymphocyte ratio
quanTIseq	Quantification of the tumor immune contexture from human RNA-seq data
ROC	Receiver operating characteristic
SD	Standard deviation
TCGA	The Cancer Genome Atlas
TIDE	Tumor Immune Dysfunction and Exclusion
TIME	Tumor immune microenvironment
TIMER	Tumor Immune Estimation Resource
TMA	Tissue microarray
TME	Tumor microenvironment
TNM	Tumor–node–metastasis
