# Supplementary material for: GPX4 Defines an Immune-Cold Phenotype and Poor Prognosis in Resected Lung Adenocarcinoma
Source: Oncol Res. 2026 Jul 16;34(8):26. doi: 10.32604/or.2026.083840 (PMC13397352; doi:10.32604/or.2026.083840)
Supplement: Supplementary file 1 [file OncolRes-34-83840-s001.zip › TSP_OR_83840-s001.pdf]

Supplementary Materials

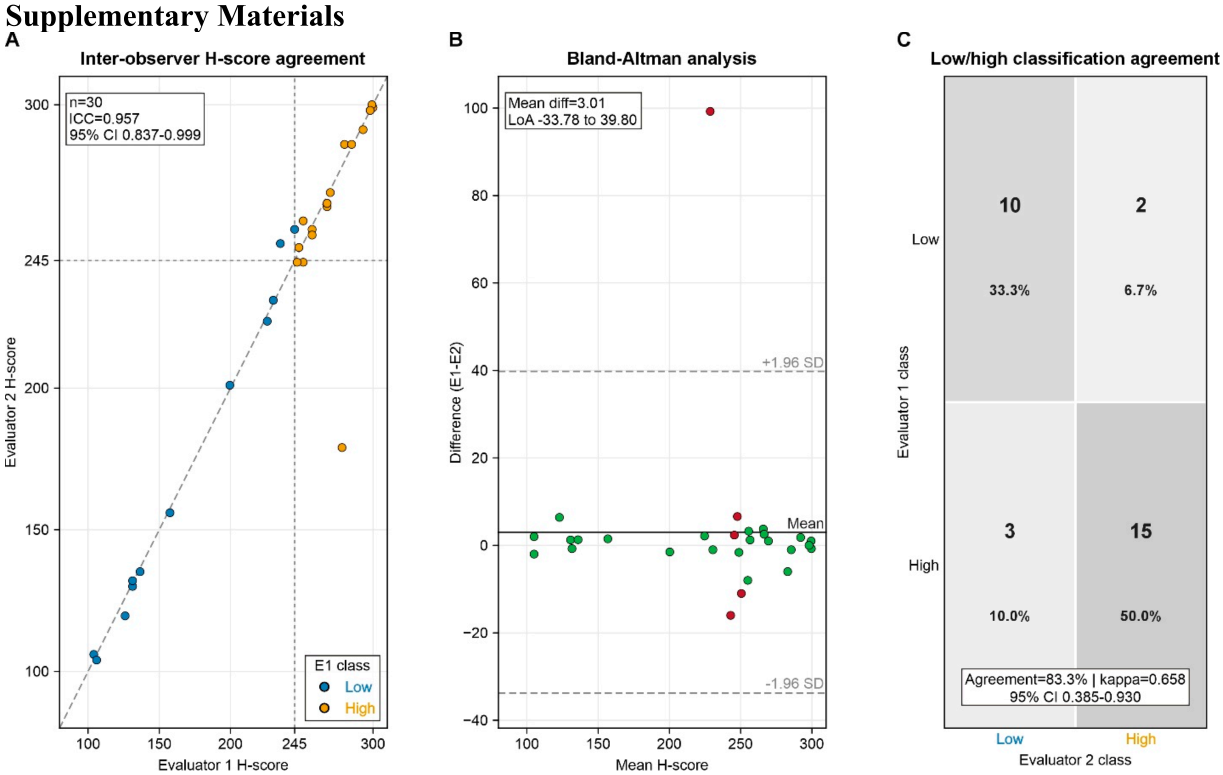

Supplementary Figure S1. Reproducibility assessment of GPX4 immunohistochemical H-score evaluation. (A) Inter-observer agreement for continuous GPX4 H-scores in 30 randomly selected cases independently reassessed by two blinded evaluators. The dashed diagonal line indicates identity between the two evaluators, and dashed horizontal and vertical lines indicate the predefined H-score cutoff of 245 used for GPX4 low/high classification. Points are colored according to evaluator 1 classification. **(B)** Bland–Altman plot assessing agreement between evaluators across the range of mean GPX4 H-scores. The solid line denotes the mean difference between evaluator 1 and evaluator 2 scores, and dashed lines indicate the 95% limits of agreement. Green points indicate cases within the 95% limits of agreement, whereas red points indicate cases outside these limits. **(C)** Agreement matrix for dichotomized GPX4 low/high classification based on the H-score cutoff of 245. Cell counts and percentages indicate concordant and discordant classifications between the two evaluators.

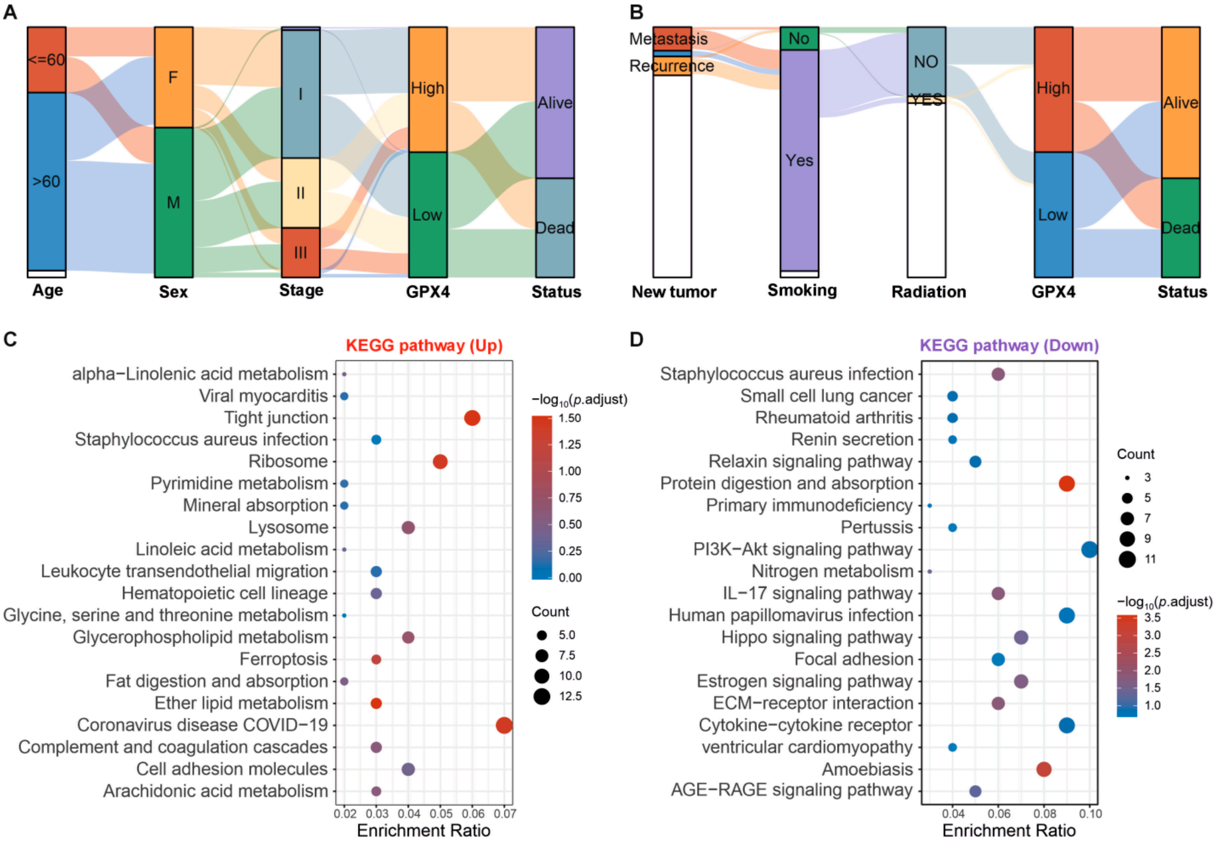

Supplementary Figure S2. Integrated clinical trajectories and pathway enrichment associated with GPX4 expression. (A) Sankey diagram illustrating the distribution of age, sex, pathological stage, GPX4 expression status and survival status across the study cohort. **(B)** Sankey diagram showing the relationships among postoperative disease events, smoking history, radiotherapy status, GPX4 expression and survival status. Flow widths are proportional to the number of patients within each category. **(C, D)** KEGG pathway enrichment analysis of genes positively **(C)** and negatively **(D)** associated with GPX4 expression. Dot size represents the number of genes mapped to each pathway, dot color indicates $-\log_{10}$-transformed adjusted p values, and the x axis shows the enrichment ratio. Statistical significance after multiple-testing correction was defined as adjusted $p<0.05$, corresponding to $-\log_{10}$(adjusted p) >1.30. In the GPX4 positively associated gene set, the coronavirus disease–COVID-19 and tight junction pathways reached this threshold, whereas the remaining upregulated pathways were interpreted as exploratory enrichment signals. In the GPX4 negatively associated gene set, several pathways reached adjusted significance, including protein digestion and absorption, amoebiasis, IL-17 signalling pathway, Hippo signalling pathway, estrogen signalling pathway, ECM–receptor interaction, cytokine–cytokine receptor interaction, focal adhesion, human papillomavirus infection, PI3K–Akt signalling pathway, relaxin signalling pathway, AGE–RAGE signalling pathway, and Staphylococcus aureus infection.

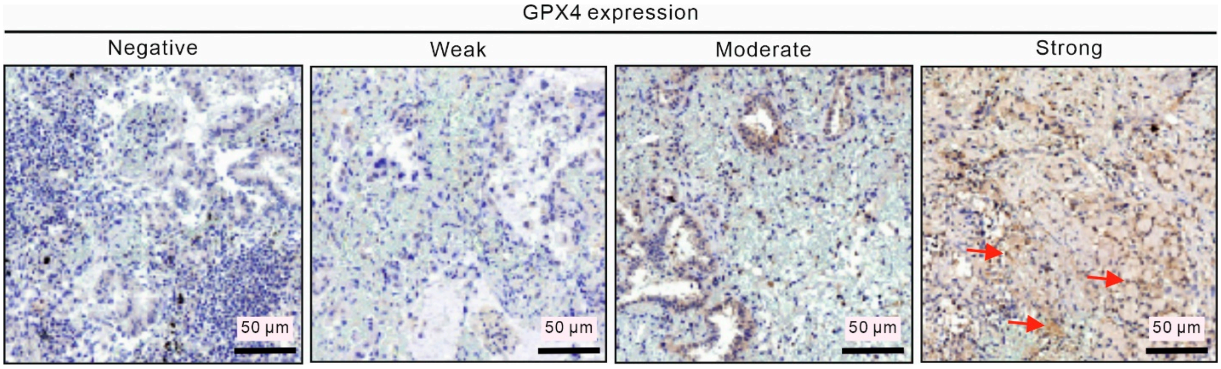

Supplementary Figure S3. Representative immunohistochemical patterns of GPX4 expression in lung adenocarcinoma. Representative immunohistochemical (IHC) micrographs illustrate the range of GPX4 staining intensity in lung adenocarcinoma tissues, including negative, weak, moderate, and strong expression. GPX4 immunoreactivity was predominantly localized in the cytoplasm of tumor cells, with progressively stronger brown chromogenic staining corresponding to increasing expression intensity. Red arrows indicate representative tumor cells with strong cytoplasmic GPX4 positivity. Sections were counterstained with hematoxylin. Scale bars, 50 μm.

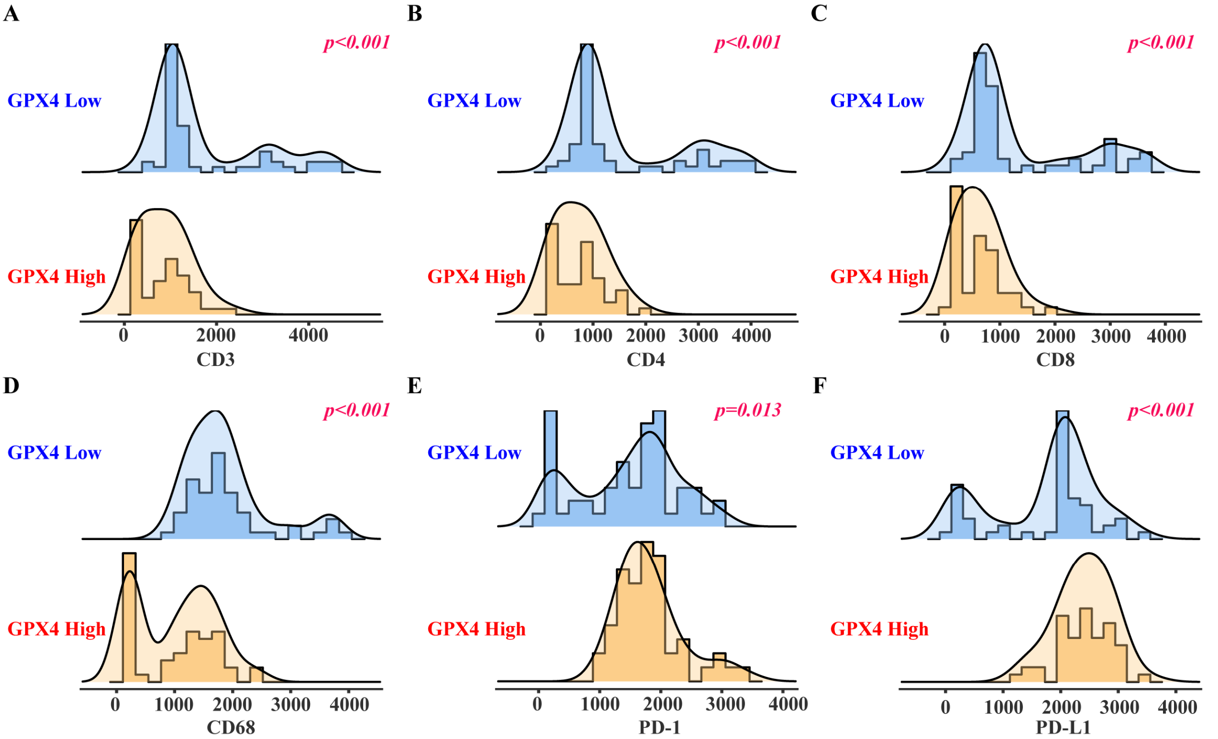

Supplementary Figure S4. GPX4 expression is associated with reduced intratumoral immune-cell infiltration and increased immune-checkpoint expression in resected lung adenocarcinoma. Distribution plots comparing intratumoral immune marker densities between the GPX4-low and GPX4-high groups for **(A)** CD3, **(B)** CD4, **(C)** CD8, **(D)** CD68, **(E)** PD-1, and **(F)** PD-L1. Tumors with high GPX4 expression showed lower densities of $CD3^+$, $CD4^+$, $CD8^+$, and $CD68^+$ cells, but higher PD-1 and PD-L1 expression. Group comparisons were performed

using the Wilcoxon rank-sum test. All *p* values were two-sided and are shown in the corresponding panels.

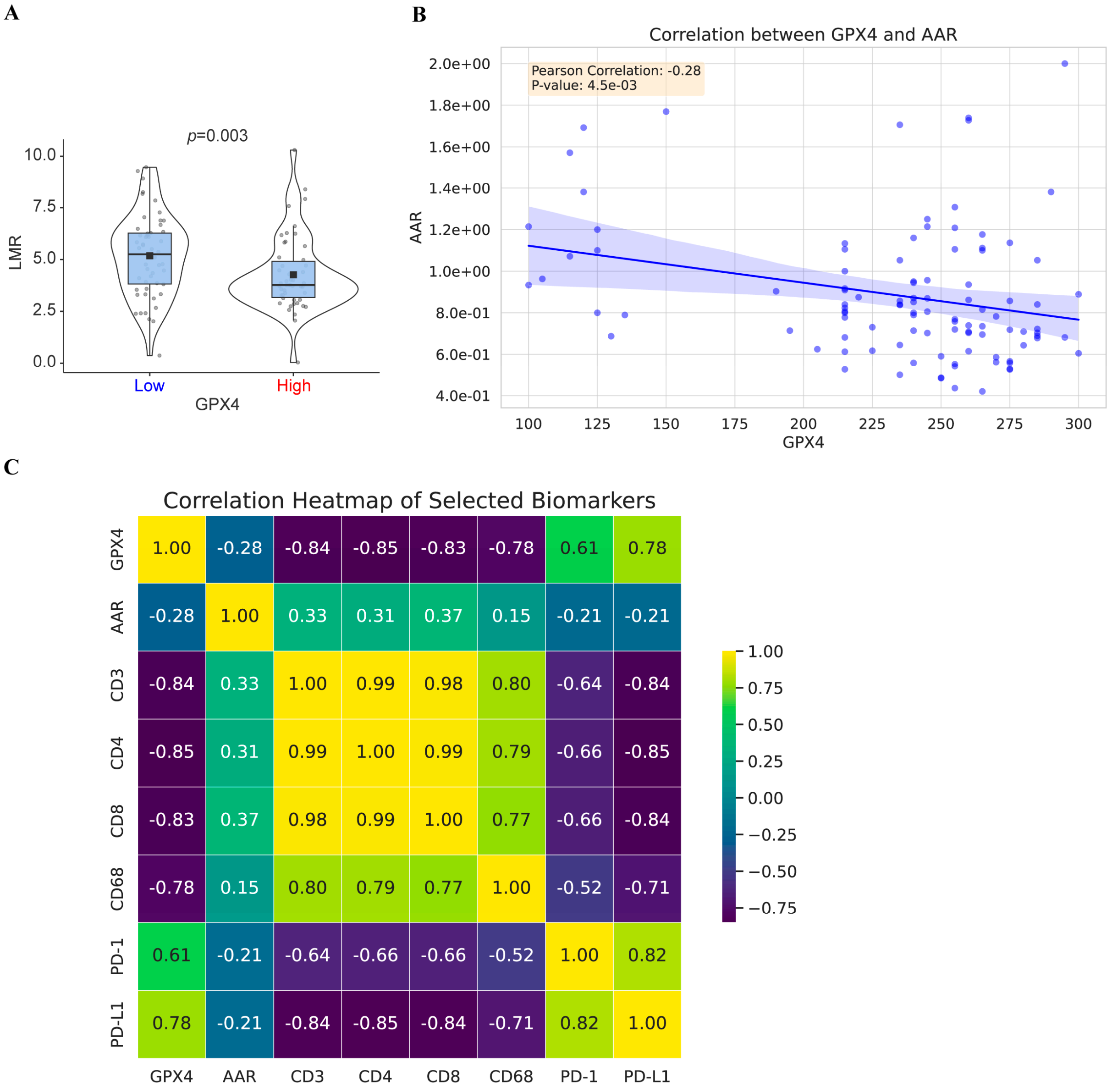

Supplementary Figure S5. Associations between GPX4 expression, systemic inflammatory indices, and immune-related biomarkers in resected lung adenocarcinoma. (A) Violin and box plots comparing the lymphocyte-to-monocyte ratio (LMR) between GPX4-low and GPX4-high tumors. The box indicates the median and interquartile range, and individual points represent patient-level values. The between-group comparison was performed using the Wilcoxon rank-sum test, with the two-sided *p* value shown in the panel. **(B)** Scatter plot showing the association between GPX4 H-score and AST-to-ALT ratio (AAR). The fitted line represents the linear regression trend, and the shaded area indicates the 95% confidence interval. Correlation was assessed using the Pearson product–moment correlation coefficient and is reported as Pearson's r with a two-sided *p* value. **(C)** Correlation heatmap integrating GPX4, AAR, intratumoral immune-

cell markers, and immune-checkpoint markers. Cell values and the color scale represent Pearson's r. All statistical tests were two-sided.

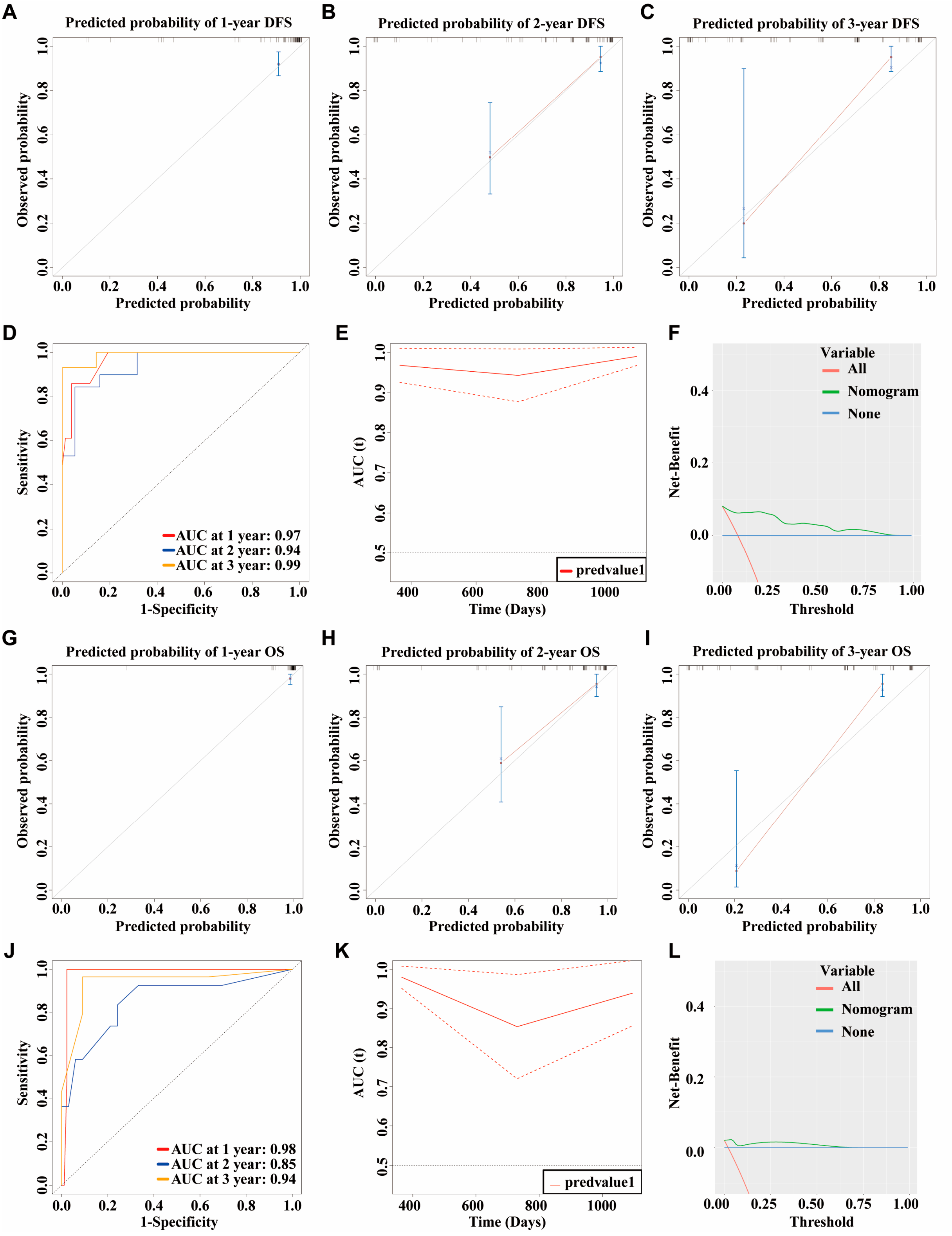

Supplementary Figure S6. Performance assessment of GPX4-based prognostic models for postoperative survival. (A–C) Calibration plots for the predicted 1-, 2-, and 3-year disease-free survival (DFS) probabilities. The x axis indicates the model-predicted probability, and the y axis indicates the observed probability. **(D)** Time-dependent receiver operating characteristic curves for predicting 1-, 2-, and 3-year DFS. **(E)** Dynamic area under the curve (AUC) estimates for the DFS model across follow-up time. The x axis is expressed in days, reflecting the original time scale used for time-dependent ROC estimation. **(F)** Decision curve analysis evaluating the net clinical benefit of the DFS nomogram across threshold probabilities. **(G–I)** Calibration plots for the predicted 1-, 2-, and 3-year overall survival (OS) probabilities, with predicted probability on the x axis and observed probability on the y axis. **(J)** Time-dependent receiver operating characteristic curves for predicting 1-, 2-, and 3-year OS. **(K)** Dynamic AUC estimates for the OS model across follow-up time, with time expressed in days. **(L)** Decision curve analysis evaluating the net clinical benefit of the OS nomogram. In the calibration plots, the diagonal line represents perfect agreement between predicted and observed probabilities. In the ROC plots, the diagonal line denotes the performance expected by chance. In the decision curve analyses, the nomogram was compared with default strategies assuming that all patients or no patients experienced the event.

Supplementary Table S1. Design and summary of the GPX4 IHC scoring reproducibility assessment.

Item	Value
Study cohort with original GPX4 H-score	104 cases
Cases selected for reproducibility reassessment	30 cases
Scoring method	H-score
Blinding	Yes
Cutoff for GPX4 classification	> 245
Original GPX4 distribution in selected cases	Low/High: 17/13
Evaluator 1 distribution	Low/High: 12/18
Evaluator 2 distribution	Low/High: 13/17
Evaluator 1 H-score, mean ± SD	231.5 ± 63.3
Evaluator 2 H-score, mean ± SD	228.4 ± 64.1
Evaluator 1 H-score, median (IQR)	251.0 (206.1–276.2)
Evaluator 2 H-score, median (IQR)	252.5 (184.5–268.1)

Note:SD, Standard Deviation; IQR，Interquartile Range.

Supplementary Table S2. Inter-observer reproducibility statistics for GPX4 H-score assessment.

Statistic	Estimate	95% CI	*p*
Intraclass correlation coefficient (ICC)	0.957	0.837–0.999	<0.001
Cohen's kappa	0.658	0.385–0.930	<0.001

Supplementary Table S3. Alternative cutoff definitions used for sensitivity analyses.

Variable	X-tile	Median cutoff	n ≤/> median	Tertile-based categories	Predefined cutoff
GPX4	≤245 *vs.* >245	245	54/50	≤235, >235–≤260, >260	>245 (median)
Age	≤60 *vs.* >60	57	60/44	≤55, >55–≤59, >59	>60 years
Ki-67	≤20, 20–40, >40	10	57/47	≤5, >5–≤20, >20	>20%
NLR	≤2.30, 2.30–3.34, >3.34	2.0932	52/52	≤1.7744, >1.7744–≤2.3946, >2.3946	>3
PLR	≤110.53, 110.53–180.81, >180.81	126.8067	52/52	≤108.6245, >108.6245–≤143.0736, >143.0736	>150
LMR	≤3.12, 3.12–4.69, >4.69	4.3751	52/52	≤3.7284, >3.7284–≤5.6187, >5.6187	≤3
CAR	≤0.03, 0.03–0.17, >0.17	0.0349	52/52	≤0.0214, >0.0214–≤0.0506, >0.0506	>0.1
APRI	≤0.10, 0.10–0.13, >0.13	0.1076	52/52	≤0.0864, >0.0864–≤0.1305, >0.1305	>0.5
AAR	≤0.69, 0.69–0.87, >0.87	0.806	52/52	≤0.7143, >0.7143–≤0.9141, >0.9141	>1.0

Note: NLR, neutrophil-to-lymphocyte ratio; PLR, platelet-to-lymphocyte ratio; LMR, lymphocyte-to-monocyte ratio; CAR, C-reactive protein-to-albumin ratio; APRI, AST to platelet ratio index; AAR, AST to ALT ratio.

Supplementary Table S4. GPX4-focused robustness analyses using alternative cutoff definitions.

Endpoint	Alternative definition	Group distribution, n/events	Contrast	HR (95% CI)	*p*
OS	Continuous variable	104/22 events	per 1-SD increase	5.42 (2.14–13.74)	<0.001
OS	Median cutoff	≤245: 54/7; >245: 50/15	>245 *vs.* ≤245	6.05 (2.28–16.00)	<0.001
OS	Tertile trend	T1: 39/6; T2: 35/8; T3: 30/8	per tertile increase	3.65 (1.94–6.86)	<0.001
OS	Tertile categories	T1: 39/6; T2: 35/8; T3: 30/8	T2 *vs.* T1	3.78 (1.17–12.22)	0.026
OS	Tertile categories	T1: 39/6; T2: 35/8; T3: 30/8	T3 *vs.* T1	13.41 (3.73–48.21)	<0.001
DFS	Continuous variable	104/32 events	per 1-SD increase	4.09 (1.95–8.55)	<0.001
DFS	Median cutoff	≤245: 54/11; >245: 50/21	>245 *vs.* ≤245	4.61 (2.11–10.10)	<0.001
DFS	Tertile trend	T1: 39/8; T2: 35/13; T3: 30/11	per tertile increase	2.65 (1.60–4.38)	<0.001
DFS	Tertile categories	T1: 39/8; T2: 35/13; T3: 30/11	T2 *vs.* T1	2.79 (1.10–7.06)	0.030
DFS	Tertile categories	T1: 39/8; T2: 35/13; T3: 30/11	T3 *vs.* T1	7.07 (2.55–19.59)	<0.001

Note:OS,Overall Survival ; DFS, Disease-Free Survival.

Supplementary Table S5. OS sensitivity analyses were performed using continuous variables and median-based dichotomization.

Variable	Continuous model	HR (95% CI)	*p*	Median cutoff	Reference/test n/events	HR (95% CI)	*p*
GPX4	per 1-SD increase	5.42 (2.14–13.74)	<0.001	>245	54/7 *vs.* 50/15	6.05 (2.28–16.00)	<0.001
Age	per 1-SD increase	1.51 (0.92–2.49)	0.101	>57	60/10 *vs.* 44/12	2.27 (0.97–5.29)	0.058
Ki-67	per 1-SD increase	1.30 (0.92–1.85)	0.135	>10	57/4 *vs.* 47/18	2.90 (0.96–8.75)	0.060
NLR	per 1-SD increase	1.55 (1.04–2.30)	0.030	>2.0932	52/2 *vs.* 52/20	7.95 (1.85–34.19)	0.005
PLR	per 1-SD increase	1.45 (1.01–2.08)	0.042	>126.8067	52/8 *vs.* 52/14	2.38 (0.97–5.83)	0.058
LMR	per 1-SD increase	0.55 (0.33–0.92)	0.024	>4.3751	52/16 *vs.* 52/6	0.33 (0.13–0.85)	0.022
CAR	per 1-SD increase	NA	NA	>0.0349	52/11 *vs.* 52/11	1.60 (0.68–3.79)	0.284
APRI	per 1-SD increase	0.91 (0.49–1.67)	0.754	>0.1076	52/11 *vs.* 52/11	1.02 (0.44–2.36)	0.965
AAR	per 1-SD increase	0.68 (0.41–1.11)	0.122	>0.806	52/11 *vs.* 52/11	0.35 (0.13–0.96)	0.041

Note:NA,not available ; NLR, neutrophil-to-lymphocyte ratio; PLR, platelet-to-lymphocyte ratio; LMR, lymphocyte-to-monocyte ratio; CAR, C-reactive protein-to-albumin ratio; APRI, AST to platelet ratio index; AAR, AST to ALT ratio.

Supplementary Table S6. OS sensitivity analyses using tertiles and clinically/predefined thresholds.

Variable	Tertile cutoffs	Tertile trend HR (95% CI)	*p*	Clinical/predefined cutoff	Reference/test n/events	HR (95% CI)	*p*
GPX4	≤235, >235–≤260, >260	3.65 (1.94–6.86)	<0.001	>245 (median)	54/7 *vs.* 50/15	6.05 (2.28–16.00)	<0.001
Age	≤55, >55–≤59, >59	1.46 (0.92–2.34)	0.110	>60 years	71/14 *vs.* 33/8	1.44 (0.60–3.45)	0.410
Ki-67	≤5, >5–≤20, >20	1.68 (0.86–3.28)	0.128	>20%	84/12 *vs.* 20/10	2.17 (0.92–5.12)	0.077
NLR	≤1.7744, >1.7744–≤2.3946, >2.3946	2.28 (1.16–4.48)	0.016	>3	82/13 *vs.* 22/9	1.70 (0.71–4.08)	0.233
PLR	≤108.6245, >108.6245–≤143.0736, >143.0736	1.38 (0.82–2.31)	0.225	>150	79/16 *vs.* 25/6	1.41 (0.54–3.70)	0.482
LMR	≤3.7284, >3.7284–≤5.6187, >5.6187	0.68 (0.40–1.16)	0.161	≤3	87/14 *vs.* 17/8	2.27 (0.94–5.51)	0.069
CAR	≤0.0214, >0.0214–≤0.0506, >0.0506	1.79 (0.98–3.27)	0.059	>0.1	85/16 *vs.* 19/6	6.56 (2.26–19.06)	<0.001
APRI	≤0.0864, >0.0864–≤0.1305, >0.1305	0.90 (0.52–1.58)	0.717	>0.5	104/22 *vs.* 0/0	–	–
AAR	≤0.7143, >0.7143–≤0.9141, >0.9141	0.53 (0.30–0.94)	0.030	>1.0	77/14 *vs.* 27/8	0.55 (0.21–1.47)	0.233

Note: NLR, neutrophil-to-lymphocyte ratio; PLR, platelet-to-lymphocyte ratio; LMR, lymphocyte-to-monocyte ratio; CAR, C-reactive protein-to-albumin ratio; APRI, AST to platelet ratio index; AAR, AST to ALT ratio.

Supplementary Table S7. Clinicopathological characteristics associated with disease-free survival (DFS) after category collapse and ridge-penalized Cox regression.

Characteristics	Category	All, n (%)	Penalized univariable HR (95% CI), *p*	Penalized multivariable HR (95% CI), *p*
Sex	Male	47 (45.2)	Reference	—
	Female	57 (54.8)	0.52 (0.26–1.05), *p*=0.068	—
Age, years	≤60	71 (68.3)	Reference	—
	>60	33 (31.7)	1.07 (0.50–2.25), *p*=0.868	—
ECOG performance status	0	62 (59.6)	Reference	—
	1–2	42 (40.4)	1.92 (0.95–3.88), *p*=0.068	—
Smoking history	Never smoker	73 (70.2)	Reference	Reference
	Smoker	31 (29.8)	2.03 (1.00–4.13), *p*=0.049	1.91 (0.74–4.96), *p*=0.181
BMI category	Underweight	13 (12.5)	Reference	—
	Normal	68 (65.4)	0.83 (0.32–2.17), *p*=0.702	—
	Overweight	23 (22.1)	0.52 (0.16–1.76), *p*=0.294	—
Pathological T stage	T0–T1	74 (71.2)	Reference	—
	T2–T3	30 (28.8)	2.12 (1.05–4.24), *p*=0.035	—
Pathological N stage	N0–N1	83 (79.8)	Reference	—
	N2	21 (20.2)	3.55 (1.71–7.37), *p*<0.001	—
Pathological stage	I	71 (68.3)	Reference	Reference
	II	12 (11.5)	2.78 (1.12–6.94), *p*=0.028	4.99 (1.53–16.30), *p*=0.008
	III	21 (20.2)	4.49 (2.04–9.90), *p*<0.001	6.10 (2.02–18.42), *p*=0.001
Tumor laterality	Left lung	64 (61.5)	Reference	—
	Right lung	40 (38.5)	1.81 (0.91–3.61), *p*=0.092	—
Histopathological subtype	Adenocarcinoma *in situ*	11 (10.6)	Reference	—
	Invasive adenocarcinoma	93 (89.4)	0.83 (0.25–2.71), *p*=0.758	—
Post-op high-risk features	None	36 (34.6)	Reference	Reference
	Single	52 (50.0)	3.43 (1.34–8.81), *p*=0.010	2.50 (0.82–7.65), *p*=0.107
	Multiple	16 (15.4)	5.03 (1.64–15.46), *p*=0.005	1.90 (0.43–8.37), *p*=0.398
CEA, μg/L	≤5	75 (72.1)	Reference	—
	>5	29 (27.9)	1.77 (0.87–3.62), *p*=0.116	—
Neutrophil count, $\times 10^9$/L	Normal (1.8–6.3)	98 (94.2)	Reference	—
	Abnormal (≤1.8 or >6.3)	6 (5.8)	0.12 (0.00–3.95), *p*=0.233	—
Lymphocyte count, $\times 10^9$/L	>1.1–3.2	91 (87.5)	Reference	—
	≤1.1	13 (12.5)	1.76 (0.73–4.26), *p*=0.208	—
Monocyte count, $\times 10^9$/L	0.1–0.6	95 (91.3)	Reference	—
	>0.6	9 (8.7)	1.98 (0.70–5.63), *p*=0.199	—
Platelet count, $\times 10^9$/L	Normal (125–350)	90 (86.5)	Reference	—
	Abnormal (≤125 or >350)	14 (13.5)	0.60 (0.15–2.40), *p*=0.472	—
NLR	≤2.30	64 (61.5)	Reference	Reference
	>2.30–3.34	25 (24.0)	2.12 (0.92–4.86), *p*=0.077	1.76 (0.65–4.74), *p*=0.266
	>3.34	15 (14.4)	4.09 (1.77–9.42), *p*<0.001	1.57 (0.29–8.66), *p*=0.602
PLR	≤110.53	40 (38.5)	Reference	Reference
	>110.53–180.81	54 (51.9)	1.13 (0.52–2.45), *p*=0.753	1.36 (0.50–3.69), *p*=0.547
	>180.81	10 (9.6)	2.69 (1.01–7.22), *p*=0.049	1.49 (0.33–6.78), *p*=0.604

	≤3.12	19 (18.3)	Reference	Reference
LMR	>3.12–4.69	38 (36.5)	0.31 (0.13–0.73), *p*=0.008	0.86 (0.27–2.74), *p*=0.792
	>4.69	47 (45.2)	0.25 (0.11–0.56), *p*<0.001	0.83 (0.23–2.96), *p*=0.773
CRP, mg/L	≤4	85 (81.7)	Reference	—
	>4	19 (18.3)	1.70 (0.69–4.21), *p*=0.249	—
Fibrinogen, g/L	Normal (>2.0–4.0)	89 (85.6)	Reference	Reference
	Abnormal (≤2.0 or >4.0)	15 (14.4)	2.84 (1.22–6.62), *p*=0.016	0.80 (0.23–2.72), *p*=0.718
Albumin, g/L	≤35	10 (9.6)	Reference	—
	>35	94 (90.4)	0.48 (0.19–1.26), *p*=0.137	—
	≤0.03	48 (46.2)	Reference	Reference
CAR	>0.03–0.17	44 (42.3)	1.23 (0.58–2.59), *p*=0.592	0.66 (0.26–1.65), *p*=0.374
	>0.17	12 (11.5)	3.02 (1.06–8.63), *p*=0.039	1.44 (0.37–5.62), *p*=0.599
ALP, U/L	Normal (>40–150)	101 (97.1)	Reference	—
	Abnormal (≤40 or >150)	3 (2.9)	0.66 (0.10–4.27), *p*=0.663	—
ALT, U/L	Normal (5–40)	98 (94.2)	Reference	—
	High (>40)	6 (5.8)	0.15 (0.00–5.97), *p*=0.315	—
AST, U/L	Normal (8–40)	101 (97.1)	Reference	—
	High (>40)	3 (2.9)	1.62 (0.23–11.38), *p*=0.627	—
	≤0.10	46 (44.2)	Reference	—
APRI	>0.10–0.13	22 (21.2)	1.81 (0.81–4.01), *p*=0.146	—
	>0.13	36 (34.6)	0.93 (0.39–2.22), *p*=0.866	—
	≤0.69	28 (26.9)	Reference	—
AAR	>0.69–0.87	36 (34.6)	1.01 (0.41–2.44), *p*=0.991	—
	>0.87	40 (38.5)	0.69 (0.28–1.69), *p*=0.410	—
γ-GGT, U/L	Normal (>11–50)	86 (82.7)	Reference	Reference
	Abnormal (≤11 or >50)	18 (17.3)	2.42 (1.03–5.68), *p*=0.043	1.24 (0.37–4.17), *p*=0.730
Ki-67 expression, %	≤20	84 (80.8)	Reference	Reference
	>20	20 (19.2)	3.00 (1.47–6.13), *p*=0.003	0.86 (0.28–2.66), *p*=0.789
GPX4 expression	Low	54 (51.9)	Reference	Reference
	High	50 (48.1)	4.42 (2.04–9.57), *p*<0.001	8.63 (2.99–24.91), *p*<0.001

Note: ECOG, Eastern Cooperative Oncology Group; BMI, body mass index; IHC, immunohistochemistry; CEA, carcinoembryonic antigen; CRP, C-reactive protein; ALP, alkaline phosphatase; ALT, alanine aminotransferase; AST, aspartate aminotransferase; γ-GGT, gamma-glutamyl transferase; NLR, neutrophil-to-lymphocyte ratio; PLR, platelet-to-lymphocyte ratio; LMR, lymphocyte-to-monocyte ratio; CAR, C-reactive protein-to-albumin ratio; APRI, AST to platelet ratio index; AAR, AST to ALT ratio.

Supplementary Table S8. Clinicopathological characteristics associated with overall survival (OS) after sparse-category consolidation and ridge-penalized Cox regression.

Characteristic	Category	All, n (%)	Univariable HR (95% CI), *p*	Multivariable HR (95% CI), *p*
Sex	Male	47 (45.2)	Reference	—
	Female	57 (54.8)	0.61 (0.25–1.53), *p*=0.293	—
Age, years	≤60	71 (68.3)	Reference	—
	>60	33 (31.7)	1.45 (0.61–3.43), *p*=0.403	—
	0	62 (59.6)	Reference	—

ECOG performance status	≥1	42 (40.4)	1.45 (0.60–3.50), p=0.412	—
Smoking history	Never smoker	73 (70.2)	Reference	—
	Smoker	31 (29.8)	1.72 (0.74–3.98), p=0.209	—
BMI category	Underweight	13 (12.5)	Reference	—
	Normal	68 (65.4)	0.57 (0.20–1.59), p=0.283	—
	Overweight	23 (22.1)	0.23 (0.05–1.10), p=0.066	—
Pathological T stage	T0–T1	74 (71.2)	Reference	—
	T2–T3	30 (28.8)	2.59 (1.12–6.01), p=0.026	—
Pathological N stage	N0–N1	83 (79.8)	Reference	—
	N2	21 (20.2)	6.04 (2.55–14.29), p<0.001	—
Pathological stage	I	71 (68.3)	Reference	Reference
	II-III	33 (31.7)	4.80 (1.90–12.18), p<0.001	4.23 (1.38–12.95), p=0.011
Tumor laterality	Left lung	64 (61.5)	Reference	—
	Right lung	40 (38.5)	1.41 (0.61–3.24), p=0.418	—
Histopathological subtype	*In situ* adenocarcinoma	11 (10.6)	Reference	—
	Invasive adenocarcinoma	93 (89.4)	0.43 (0.10–1.94), p=0.274	—
Post-op high-risk features	None	36 (34.6)	Reference	—
	Any high-risk feature	68 (65.4)	6.00 (1.53–23.61), p=0.010	—
CEA, μg/L	≤5	75 (72.1)	Reference	—
	>5	29 (27.9)	1.64 (0.70–3.86), p=0.254	—
Neutrophil count, $\times 10^9$/L	Normal (1.8–6.3)	98 (94.2)	Reference	—
	Abnormal	6 (5.8)	0.24 (0.00–12.81), p=0.480	—
Lymphocyte count, $\times 10^9$/L	≤1.1	13 (12.5)	Reference	—
	1.1-3.2	91 (87.5)	0.66 (0.22–1.94), p=0.446	—
Monocyte count, $\times 10^9$/L	0.1-0.6	95 (91.3)	Reference	—
	>0.6	9 (8.7)	1.14 (0.33–3.90), p=0.835	—
Platelet count, $\times 10^9$/L	Normal (125–350)	90 (86.5)	Reference	—
	Abnormal	14 (13.5)	1.56 (0.37–6.69), p=0.547	—
NLR	≤2.3	64 (61.5)	Reference	Reference

	2.3-3.34	25 (24.0)	3.41 (1.20–9.74), p=0.022	2.11 (0.56–7.97), p=0.271
	>3.34	15 (14.4)	3.63 (1.18–11.12), p=0.024	2.22 (0.59–8.43), p=0.240
PLR	≤110.53	40 (38.5)	Reference	—
	110.53-180.81	54 (51.9)	2.45 (0.89–6.80), p=0.084	—
	>180.81	10 (9.6)	4.33 (1.30–14.42), p=0.017	—
LMR	≤3.12	19 (18.3)	Reference	—
	3.12–4.69	38 (36.5)	0.64 (0.24–1.67), p=0.359	—
	>4.69	47 (45.2)	0.28 (0.09–0.81), p=0.019	—
CRP, mg/L	≤4	85 (81.7)	Reference	Reference
	>4	19 (18.3)	6.21 (2.18–17.72), p<0.001	6.91 (2.00–23.81), p=0.002
Fibrinogen, g/L	Normal (2.0–4.0)	89 (85.6)	Reference	—
	Abnormal	15 (14.4)	5.10 (1.90–13.64), p=0.001	—
Albumin, g/L	≤35	10 (9.6)	Reference	—
	>35	94 (90.4)	0.38 (0.14–1.03), p=0.058	—
CAR	≤0.03	48 (46.2)	Reference	—
	0.03-0.17	44 (42.3)	1.52 (0.59–3.92), p=0.383	—
	>0.17	12 (11.5)	9.03 (2.73–29.86), p<0.001	—
ALP, U/L	Normal (40–150)	101 (97.1)	Reference	—
	Abnormal	3 (2.9)	0.70 (0.10–4.67), p=0.709	—
ALT, U/L	5–40	98 (94.2)	Reference	—
	>40	6 (5.8)	0.44 (0.00–45.42), p=0.731	—
AST, U/L	8–40	101 (97.1)	Reference	—
	>40	3 (2.9)	8.38 (0.85–82.89), p=0.069	—
APRI	≤0.1	46 (44.2)	Reference	—
	0.1-0.13	22 (21.2)	1.04 (0.39–2.71), p=0.944	—
	>0.13	36 (34.6)	0.66 (0.23–1.91), p=0.443	—
AAR	≤0.69	28 (26.9)	Reference	—
	0.69-0.87	36 (34.6)	0.49 (0.17–1.44), p=0.195	—

	>0.87	40 (38.5)	0.21 (0.07–0.69), p=0.009	—
γ-GGT, U/L	Normal (11-50)	86 (82.7)	Reference	Reference
	Abnormal	18 (17.3)	3.56 (1.35–9.43), p=0.011	1.68 (0.48–5.86), p=0.413
Ki-67 expression, %	≤20	84 (80.8)	Reference	Reference
	>20	20 (19.2)	2.10 (0.90–4.91), p=0.088	0.71 (0.27–1.91), p=0.503
GPX4 expression	Low	54 (51.9)	Reference	Reference
	High	50 (48.1)	5.97 (2.30–15.51), p<0.001	6.94 (2.44–19.74), p<0.001

Note: ECOG, Eastern Cooperative Oncology Group; BMI, body mass index; IHC, immunohistochemistry; CEA, carcinoembryonic antigen; CRP, C-reactive protein; ALP, alkaline phosphatase; ALT, alanine aminotransferase; AST, aspartate aminotransferase; γ-GGT, gamma-glutamyl transferase; NLR, neutrophil-to-lymphocyte ratio; PLR, platelet-to-lymphocyte ratio; LMR, lymphocyte-to-monocyte ratio; CAR, C-reactive protein-to-albumin ratio; APRI, AST to platelet ratio index; AAR, AST to ALT ratio; HR, hazard ratio.

Supplementary Table S9. Event-per-variable assessment and revised parsimonious multivariable Cox model for disease-free survival (DFS).

**Component**	**Definition or contrast**	**Events**	**Free parameters**	**EPV**	**HR (95% CI)**	***p***
Endpoint definition	Recurrence/progression before death or death before last follow-up	32	—	—	—	—
Revised primary model	Pathological stage + GPX4 expression	32	2	16	—	—
Pathological stage	Stage II–III *vs.* stage I	32	1	—	7.88 (3.63–17.08)	<0.001
GPX4 expression	High *vs.* low	32	1	—	9.63 (4.06–22.88)	<0.001
Initial full model	11 covariates with multilevel terms	32	16	2	—	—
Broader penalized sensitivity model	Expanded clinically screened model after category consolidation	32	17	1.9	—	—

Note:EPV, events per variable; HR, hazard ratio.

Supplementary Table S10. Event-per-variable assessment and revised parsimonious multivariable Cox model for overall survival (OS).

Component	Definition or contrast	Events	Free parameters	EPV	HR (95% CI)	*p*
Endpoint definition	Death from any cause	22	—	—	—	—
Revised primary model	Pathological stage + GPX4 expression	22	2	11	—	—
Pathological stage	Stage II–III *vs.* stage I	22	1	—	5.41 (2.07–14.13)	<0.001
GPX4 expression	High *vs.* low	22	1	—	6.08 (2.34–15.82)	<0.001
Initial full model	Five covariates with multilevel terms	22	8	2.8	—	—
Broader penalized sensitivity model	Expanded clinically screened model after category consolidation	22	7	3.1	—	—

Note:EPV, events per variable; HR, hazard ratio.

Supplementary Table S11. Bootstrap internal validation of GPX4-based prognostic models for disease-free survival (DFS) and overall survival (OS).

**Endpoint**	**Apparent C-index**	**Optimism**	**Bias-corrected C-index**	**Bias-corrected calibration slope**
DFS	0.799	0.017	0.782	0.964
OS	0.838	0.012	0.826	0.937
